# A Chemical Approach to the Synthesis of an Antitumoral Pt-Based Drug Molecule Using a Protein-Ligand Interaction as a Carrier System

**DOI:** 10.1021/acsomega.5c13604

**Published:** 2026-04-22

**Authors:** Ulises Galindo-García, Rodrigo Flores-Manzo, Monserrat Yesenia Garrido-Santos, Alan Juárez-Barragán, Josué Valdés-García, Diego Martínez-Otero, Andrey Fabricio Ziem Nascimento, Mayra Cuéllar-Cruz, Enrique García-Hernandez, Alejandro Dorazco-González, Abel Moreno

**Affiliations:** † Instituto de Química, 7180 Universidad Nacional Autónoma de México, Avenida Universidad 3000. Colonia UNAM, Mexico City 04510, Mexico; ‡ Instituto de Investigaciones en Materiales, Universidad Nacional Autónoma de México, Avenida Universidad 3000. Colonia UNAM, Mexico City 04510, Mexico; § Center for Research in Sustainable Chemistry (CCIQS) UAEMéx-UNAM, Toluca 50200, Mexico; ∥ Brazilian Synchrotron Light Laboratory (LNLS), Brazilian Center for Research in Energy and Materials (CNPEM), Campinas, Sao Paulo 13083-970, Brazil; ⊥ Departamento de Biología, División de Ciencias Naturales y Exactas, Universidad de Guanajuato, Campus Guanajuato, Noria Alta S/N, Col. Noria Alta, Guanajuato, Guanajuato 36050, México

## Abstract

In this paper, we explore different strategies that can serve as models for both the design and delivery of candidate antitumoral molecules intended for cancer therapy. For example, a novel platinum-based compound, called 1.Cl, was synthesized, and its interaction with the iron-transport protein transferrin, proposed as a potential targeted drug delivery system, was studied. The synthesis of 1.Cl and its interaction with transferrin were studied using a multimethodological approach based on spectroscopic, thermodynamic, and solid-state methods, including UV–vis absorption and emission spectroscopy, isothermal titration calorimetry, differential scanning calorimetry, and protein crystal X-ray diffraction. The successful synthesis and design of 1.Cl, a compound with structural and chemical properties that may enable antitumoral activity, along with the study of its interaction with a native iron-transport protein, provide a promising foundation for the design of potential cancer drugs. The results indicate that 1.Cl interacts with transferrin effectively in solution, supporting the hypothesis that this protein can be used as a selective delivery system to enhance cytotoxicity in malignant cells while reducing side effects.

## Graphical abstract

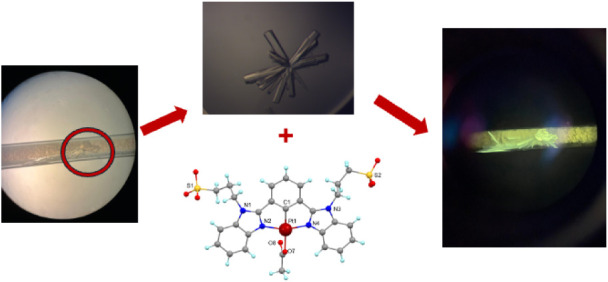

## 1 Introduction

Nowadays, there is particular interest in developing new synthetic molecules that are soluble, cell-membrane-permeable, and readily absorbed by the human body to treat diseases such as cancer. For decades, metal-based complexes have been studied as anticancer drugs; scarce contributions have come from a crystallographic perspective, reviewing the topic^1^ or, particularly, regarding metal binding to human ubiquitin.^2^ Cancer remains one of the most significant global public health challenges and originates from uncontrolled cellular proliferation resulting from regulatory and self-repair mechanism failure.^3^ Conventional treatment strategies include immunotherapy, targeted therapy, gene therapy, and chemotherapy. Many chemotherapeutic agents exhibit limited selectivity, systemic toxicity, and the potential to induce drug resistance, highlighting the growing need to develop new therapeutic agents and drug-delivery strategies that serve as treatments for this disease.

Among metal-based anticancer molecules, platinum compounds remain as the most studied and used.^4^ Cisplatin, the first approved platinum-based cancer treatment,^5^ exerts its cytotoxic activity through coordination to DNA, causing structural distortion that inhibits replication and triggers apoptosis, leading to cell cycle arrest.^6,7^ However, the use of this drug is associated with multiple side effects in the liver, kidneys, and heart,^8^ as well as the emergence of cell resistance mechanisms that inhibit the biological activity of the platinum derivative.^6^ These limitations have driven the development of alternative platinum-derived anticancer molecules as well as alternative delivery strategies aimed at enhancing selectivity while reduced toxicity. It has been studied that, in humans, cisplatin circulates in the blood largely bound to plasma proteins, predominantly serum albumin^9,10^ and, to a lesser extent, transferrin;^11^ interactions with hemoglobin have also been described, suggesting that red blood cells may contribute to its distribution. Cellular uptake of cisplatin is mediated in part by the copper transporter CTR1.^12^

A promising approach involves using endogenous protein carriers to improve drug targeting. Transferrin, an iron-transporting protein, is of particular interest as its receptor (TfT) expression is upregulated in numerous cancer cell lines, including lung, breast, and gastric tumors.^13^ This overexpression can facilitate the internalization of the transferrin-drug complex, which results in an enhanced cellular uptake compared with free drugs. Additionally, solid-state studies indicate that cisplatin binding does not compete with the native iron-binding sites,^14,15^ supporting the feasibility of this approach, as it is required for the transferrin to be loaded with iron to be recognized by the TfT.

The present work focuses on the design and evaluation of a novel Pt^2+^-based compound (1.C1) with high hydrophilicity and low cytotoxicity as an alternative to FDA-approved drugs, which in most cases result in being highly expensive. This study offers an alternative framework for the rational development of more efficient candidate treatment molecules. The system was designed to retain sufficient affinity to transferrin to enable transport while maintaining labile enough to be released by chemical stimuli. In particular, the compound is designed to trigger the release of the compound due to the pH change associated with transferrin receptor-mediated endocytosis. The study aims to evaluate its interaction with transferrin as a potential delivery system through thermodynamic, spectroscopic, and crystallization approaches.

## 2 Experimental Section

### 2.1 Reagents and Protein

#### 2.1.1 Reagents

All general reagents (Sigma-Aldrich, St. Louis, Missouri) were used without further purification; all were molecular biology grade or equivalent. The water used was of molecular biology grade, with a resistivity of over 18.2 MΩ·cm. The samples were weighed using a DV215CD Discovery XP6 microbalance (Mettler Toledo International Inc., Columbus, Ohio). The buffer solutions were filtered through a 0.45 μm filter and sonicated before use. All experiments were performed at room temperature unless otherwise specified.

#### 2.1.2 Protein

*Holo-*Transferrin human plasma (*h*Tf) (Merck KGaA, Darmstadt) and *apo*-Transferrin human plasma (*a*Tf) (Sigma-Aldrich, St. Louis, Missouri) purity assay ≥95%. Fresh, filtered protein solutions were used (a cellulose low-binding filter with a pore size of 0.45 μm). For crystallization experiments, protein purification was achieved using Fast Protein Liquid Performance ÄKTA pure (Cytiva/GE Healthcare Bio-Sciences, Inc., Marlborough, Massachusetts) by Size-Exclusion Chromatography using a 5 mL column HiTrap desalting packed with sepharose and performed in HEPES (15 mM HEPES, 20 mM NaHCO_3_ and 50 mM NaCl) pH 7.4 with a flow rate of 1 mL·min^–1^ (Figure S1) then concentrated by centrifugation in 0.5 cm^3^ Amicon Ultra Centrifugal Filter, 3 kDa MWCO regenerated cellulose membrane at 3,000 × *g* in 15 min cycles at 277 K.

### 2.2 Platinum-Based Agent

#### 2.2.1 Chemical Synthesis

##### 2.2.1.1 Synthesis of L^0^, 1,3-bis­(1*H*-Benzo­[*d*]­imidazol-2-yl)­benzene

L^0^ was synthesized according to the reported procedure.^16^

##### 2.2.1.2 Synthesis of L^1^, 3,3′-(1,3-phenylenebis­(1*H*-benzo­[*d*]­imidazole-3-ium-2,1-diyl))­bis­(propane-1-sulfonate)

Ligand L^1^ was performed by a modified procedure reported previously.^17^ L^0^ (150.7 mg, 0.48 mmol) was treated with Cs_2_CO_3_ (397.8 mg, 1.22 mmol) in 5 mL of dry DMF. The mixture was stirred for 1 h at room temperature under a N_2_ atmosphere. After, a solution of 1,3-propane sultone (127.1 mg, 1.04 mmol) in 1 mL of dry DMF was added, and the reaction was stirred overnight at room temperature under an atmosphere of dinitrogen. Thus, the reaction mixture was filtered through Celite 503, and the solvent was removed by evaporation under reduced pressure. After the mixture was dried, a solution of HCl (90 μL, 37%) in 7 mL of water was added, and the mixture was stirred for 30 min, obtaining a white crystalline solid. The solid was collected by filtration, washed with 5 mL of cold water, and dried under a vacuum. Yield: 254.4 mg, 83%. ^1^H NMR (300 MHz, 298 K, DMSO-*d*_6_): δ (ppm) 8.42 (s, 1H), 8.32 (d, *J* = 8.0 Hz, 2H), 8.23–8.12 (m, 2H), 8.07 (t, *J* = 7.9 Hz, 1H), 7.99–7.88 (m, 2H), 7.73–7.60 (m, 4H), 4.75 (t, *J* = 8.3 Hz, 4H), 2.70 (t, *J* = 6.2 Hz, 4H), 2.30 (dq, *J* = 14.7, 5.7 Hz, 4H). ^13^C NMR (126 MHz, 298 K, DMSO-*d*_6_): δ (ppm) 148.67, 134.41, 132.71, 131.50, 130.74, 126.62, 126.22, 124.07, 115.07, 113.66, 47.80, 44.89, 25.08. IR-ATR (cm^–1^): ν = 3371.81 (m), 2521.27 (m), 1460.88 (m), 1216.09 (s), 1149.89 (s), 1034.78 (s), 762.54 (s), 514.66 (s).

ESI-MS (*m*/*z*): [L^1^]^+^; 554.7 [L^1^ + H^+^]^+^ Elem. Anal. Calcd for C_26_H_26_N_4_O_6_S_2_ (554.64 g·mol^–1^): C, 56.30; H, 4.73; N, 10.10; S, 11.56; Found: C, 55.68; H, 4.65; N, 10.02; S, 11.77.

##### 2.2.1.3 Synthesis of 1.Cl

A mixture of L^1^ (64.4 mg, 0.1161 mmol) and K_2_[PtCl_4_] (53 mg, 0.127 mmol) in acetic acid (20 mL) was stirred for 24 h at 393 K under a N_2_ atmosphere. After the solvent was cooled and removed at reduced pressure, the residue was dissolved in 50 mL of hot CH_3_OH and filtered through Celite 503. Subsequently, the solvent was removed by reduced pressure, and a yellow solid was acquired. The yellow solid was stirred for 10 min in 5 mL of CH_3_OH, and the solution was drawn off. The solid was dried under a vacuum. Yield: 82.6 mg, 82%.

^1^H NMR (300 MHz, 298 K, DMSO-*d*_6_): δ (ppm) 8.85–8.76 (m, 2H), 8.02 (d, *J* = 7.8 Hz, 2H), 7.95–7.86 (m, 2H), 7.49–7.34 (m, 5H), 4.94 (t, *J* = 6.9 Hz, 4H), 2.66 (t, *J* = 6.8 Hz, 4H), 2.22 (q, J = 7.09, 4H). ^13^C NMR (76 MHz, 298 K, DMSO-*d*_6_): δ (ppm) 161.10, 140.52, 134.28, 131.27, 125.42, 124.22, 123.82, 123.61, 117.59, 111.56, 47.94, 43.45, 26.13. IR-ATR (cm^–1^): ν = 3371.81 (m), 2521.27 (m), 1460.88 (m), 1216.09 (s), 1149.89 (s), 1034.78 (s), 762.54 (s), 514.66 (s). ESI-MS (*m*/*z*): [1.Cl]^−^; 805.1 corresponding to [1.Cl – 2 K^+^ + Na^+^]^−^. Elem. Anal. Calcd For C_26_H_23_ClK_2_N_4_O_6_PtS_2_ (860.34 g·mol^–1^): C, 36.30; H, 2.69; N, 6.51; S, 7.45; Found: C, 35.92; H, 3.10; N, 6.41; S, 7.43.

### 2.3 Single Crystal X-ray Diffraction (SC-XRD)

Data for 1.Aco were collected on a Bruker APEX II CCD Diffractometer (Toluca, State of México, México) at 100 K, using *M*_*o*_ *–K*_*a*_ radiation (*k* = 0.71073 Å) from an Incoatec ImuS sources and Helios optic monochromator.^18^ Suitable crystals were coated with hydrocarbon oil, picked up with a nylon loop, and mounted in the cold nitrogen stream (100 K) of the diffractometer. Frames were collected using ω scans and integrated with SAINT. Multiscan absorption correction (SADABS) was applied.^19^ The structures were solved by direct methods and refined using full-matrix least-squares on *F* ^2^ with SHELXL-2018 ^20^ using the SHELXLE GUI.^21^

The solvent molecules (CH_3_OH), coordinated to the Mg^2+^ atoms, exhibit an occupational disorder. This was modeled using the SIMU, RIGU, and SAME restraints implemented in the SHELXLE GUI.^21^ The occupancies were refined using a free variable, resulting in ratios of 69:31 and 87:13 for the two CH_3_OH molecules.

The potassium counterion also displays occupational disorder at four different positions. This was modeled using the SIMU, RIGU, and ISOR restraints in SHELXLE.^21^ The occupancies were initially refined using a free variable and then constrained to sum to unity, yielding final occupancy ratios of 32:22:21:24, respectively.

Crystallographic data for crystal structure have been deposited with the Cambridge Crystallographic Data Centre as Supporting Information no. CCDC 2483797.

### 2.4 Cytotoxicity of 1.Cl

Cytotoxic activity of 1.Cl as a growth inhibitor against cancer cells was evaluated as previously reported^16^ using prostate (PC-3), leukemia (K562), colon (HCT-15), breast (MCF-7), lung (SKLU1), and brain (U-251) tumoral cellular lines. In addition, COS-7 nontumoral cells were tested. The inhibitor’s concentration used was 10 μM in water. The inhibition relation was determined by a primary screening using the sulforhodamine B protocol (SRB),^22,23^ in a TC20 Automated Cell Counter (Bio-Rad, Hercules, California). PC-3, K562, HCT-15, MCF-7, and SKLU1 cell lines were supplied by the National Cancer Institute (USA), while the Cancer Institute of México donated COS-7. The cell lines were cultured in RPMI-1640 medium (Gibco) supplemented with 10% fetal bovine serum, Penicillin-Streptomycin solution 100× (Corning) and 1% nonessential amino acids (Gibco). According to the protocol, the viability of the used cells exceeds 95%, as determined by using trypan blue.

### 2.5 Crystallization *a*Tf-1.Cl

A mixed solution of 1.0 mM 1.Cl and 20 mg·mL^–1^ *a*Tf solutions, freshly prepared in HEPES buffer, was filtered through Nalgene 4 mm 0.45 μm and was in turn mixed 1:1 with citrate solution (0.2 M ammonium citrate tribasic, pH 7.0, 20% w/v polyethylene glycol 4,000) as the precipitant agent (PA).

*Vapor diffusion*: In the MRC 2 Well Crystallization Plate (Laboratory of Molecular Biology, Cambridge), 2.0 μL of the mix was placed in each well reservoir, A1, and 50 μL of precipitant agent in the A1 diffusion reservoir. Then, 1.0 μL of A1 is mixed with PA 1:1 to dilute and placed in A2, and the dilution process is continued until the box line is filled. At the end, the box was sealed and stored. *Hanging-drop*: 5 μL of the mix was in the center of tap A1 of an EasyXtal 15-Well Plates (QIAGEN, Maryland), and 50 μL of precipitant agent was in the diffusion reservoir. Then 2.5 μL of A1 was mixed with 2.5 μL of PA, 1:1, and collocated in the B1 tap, repeating the dilution process until the box line was filled. Screw every tap, and store. The crystallization systems were placed at a controlled temperature of 291.1 K without disturbance. *Diffusion-controlled transport processes by the gel capillary*: 0.66% ultrapure low-melting agarose was mixed with 20 mg·mL^–1^ *a*Tf solution 1:1 and placed into an HR6–160/0.5 mm quartz capillary (length 80 mm; outer Φ 0.5 mm; wall thickness 0.01 mm; Hampton Research, Aliso Viejo, California), and the lower side was sealed with plasticine, and the upper side was filled with 20 μL of agarose, sealed, and stored. After 4 weeks, the superior seal was removed, refilled with 20 μL of 1.Cl 1.0 mM in HEPES buffer, and sealed again.

### 2.6 X-ray Diffraction at the Synchrotron Facility

The X-ray diffraction data were collected at 100 K on the MANACÁ beamline at the Sirius synchrotron (LNLS, CNPEM, Campinas, Brazil) using a PILATUS 2M detector (Dectris Ltd.) and an X-ray wavelength of 0.97718 Å. The data were collected using an oscillation of 0.1° per image and a full (360°) ω-rotation scan. The diffraction images were processed using XDS.^24^ Unfortunately, only low-resolution data sets were obtained, making structural determination impossible. The data collection statistics are listed in Table 1.

**Table 1:** Data Collection Statistics^a^

	Transferrin
Wavelength (Å)	0.97718
Space group	*C*2
Cell dimensions	
a, b, c (Å)	218.32, 85.84, 101.67
α, β, γ (°)	90, 113.71, 90
Unique reflections	28801 (4521)
Resolution (Å)	57.56–3.19 (3.38–3.19)
Rmeas (%)	16.9 (85.6)
Mean I/σ(I)	32.69 (1.90)
C*C*_1/2_	99.6 (57.9)
Completeness (%)	99.3 (97.3)
Redundancy	6.05 (5.09)
Wilson B-factor (Å^2^)	72.26

a Values in parentheses are for the highest resolution shell.

### 2.7 Differential Scanning Calorimetry (DSC)

DSC measurements were performed using a MicroCal VP-Capillary DSC instrument (Malvern Panalytical Inc., USA), as described previously.^25^ Buffer–buffer baselines were obtained under the same experimental conditions and subtracted from the sample traces. All results presented were obtained at a scan rate of 60 K·h^–1^. The endotherms were fitted to independent transition-unfolding models programmed in MicroCal Origin version 7 software (OriginLab Corporation, Northampton, Massachusetts).

### 2.8 Fluorescence and UV–vis Spectra (FL, UV–vis)

An Agilent Cary Eclipse Fluorimeter (Agilent Technologies, Santa Clara, California), coupled with a 75 W xenon short-arc lamp and an Agilent Cary 100 spectrophotometer, was used to acquire fluorescence and UV–vis spectra, respectively. Both were equipped with a thermostated single-cell holder at 298 K (±0.1). FL titrations were performed in a quartz cuvette 2.0 cm^3^ containing 5 μM of 1.Cl, with *a*Tf at 11.28 μM in the same buffer. The system was excited at λ = 360 nm and 670 V; meanwhile, absorbance in UV–vis titration was registered from 250 to 500 nm in the quartz cuvette containing 1.Cl 45 μM titrated with *a*Tf 9.63 μM. The solutions were prepared in HEPES at pH 7.4.

### 2.9 Isothermal Titration Calorimetry (ITC)

ITC measurements were carried out using a MicroCal iTC200 instrument (GE Healthcare, Northampton, Massachusetts) as described previously.^26^ The titration schedule consisted of 20 consecutive injections of ligand with a 5 min interval between injections, using a stirring rate of 750 rpm. The dilution heat of the ligand was obtained by adding the ligand to a buffer solution under identical conditions and the same injection schedule used with the protein sample. All samples were degassed for 10 min prior to the experiment. Binding parameters were determined by using an identical and independent binding site model:

$$Q=\frac{nM_{t}\Delta H_{b}V_{O}}{2}\left[1+\frac{L_{t}}{nM_{t}}+\frac{1}{nK_{b}M_{t}}-\sqrt{{\left(1+\frac{L_{t}}{nM_{t}}+\frac{1}{nK_{b}M_{t}}\right)}^{2}-\frac{4L_{t}}{nM_{t}}}\right]$$

where *Q* is the normalized heat evolved per mol of ligand at the end of the *i*th injection, *K*_*b*_ is the binding constant, Δ*H*_*b*_ is the enthalpy change, *n* is the stoichiometry, *V*_*o*_ is the working volume of the cell, and *L*_*t*_ and *M*_*t*_ are the total ligand and macromolecule concentrations, respectively. The heat released in the *i*th injection is

$$\Delta Q_{i}=Q_{i}+\frac{v_{i}\left(Q_{i}+Q_{i-1}\right)}{2V_{O}}-Q_{i-1}+q_{dil}$$

where *v*_*i*_ is the aliquot volume added at injection *i* and *q*_*dil*_ is a fitting term introduced to account for experimentally uncorrected dilution heat effects. The nonlinear regression was carried using the MicroCal Origin v7.

### 2.10 Structural Characterization of the Chemical Interaction via X-ray Crystallography and Conventional Methods

Single-crystal X-ray irradiation is a powerful technique for determining the 3D crystallographic structure of biological macromolecules at very high resolution, provided that well-diffracting crystals can be obtained for a specific target biomolecule. However, X-ray diffraction is not the only method for obtaining the 3D structure of biological macromolecules. Other techniques, such as Nuclear Magnetic Resonance (NMR) or computational modeling of biomolecules (which predict their 3D structure through homology and similarity), can yield valuable information. A technique that provides additional low-resolution structural information is small-angle X-ray scattering (SAXS). Still, it can be used to estimate the chemical and physical properties of proteins in solution (e.g., protein–protein or protein–solvent interactions). In addition to these possibilities, X-ray diffraction is the only method that can determine the ligand’s position with high accuracy. The unsolved problem has been the feasibility of obtaining high-quality crystals of human transferrins that diffract to resolutions of less than 2.0 Å. The last possibility was to employ counter-diffusion methods in a gel, thereby allowing the ligand to be incorporated into the crystalline structure at a lower level.

The counter-diffusion technique is based on the principle of mass transport governed by diffusion, facilitated by experimentation within a chamber. Diffusion provides a homogeneous distribution of supersaturation in the crystallization solution, which, in turn, allows molecules to find more favorable positions within the crystal lattice, resulting in a stable crystal. Capillary tubes are used for counter-diffusion experiments due to their elongated shape and, primarily, their definite diameter, which provides a restricted space in which convection is reduced, allowing crystals to grow over their entire internal surface area. Crystal nucleation and growth occur when the precipitating agent meets the protein solution at the liquid–liquid interface, forming a supersaturation gradient. One advantage of counter diffusion over conventional methods is that it facilitates screening of various conditions within the same experiment, thanks to differences in the diffusion coefficients of the components of the precipitating agent. Besides being practical, it is efficient, because it requires a small amount of protein solution and ligand.

## 3 Results and Discussion

### 3.1 Synthesis, Crystal Structure, and Photophysical Properties of 1.Cl

Cyclometalated Pt^2+^-N̂ĈN complexes with square planar geometry and d^8^ electronic configuration (N̂ĈN= derivatives of 1,3-bis­(phenylbenzimidazolyl)­benzene) are typically brightly green-emitting compounds with long-lived lifetimes at the millisecond level.^27–32^ These organometallic Pt^2+^ complexes have attracted attention due to their outstanding property to undergo supramolecular self-assemblies by reversible interactions such as metallophilic interactions and π···π stacking contacts^33^ as well as their application in photochromism,^31^ OLEDs,^34–36^ sensing of ions,^37,38^ catalysis,^39^ and visible-light harvesting.^40^ However, their interaction with proteins and DNA remains largely unexplored.

Reports in the context of medicinal chemistry have shown that analogous coordination Pt^2+^-N'^'N'^'N complexes (N'^'N'^'N = terpy) are able to bind DNA and proteins in a micromolar concentration range.^41–44^ One advantage of organometallic complexes of the Pt^2+^-N'^'C'^'N type is the strong trans influence of the central anionic phenyl ligand, which, in principle, should favor reversible chemical equilibria in solution and increase affinity through the substitution of the chloride anion by stronger coordinating species such as amino acids.^38^

Taking this into account, we surmised that a hydrostable Pt^2+^complex with affinity to transferrin in water could be achieved by using an organometallic Pt^2+^-N'^'C'^'N complex bearing hydrophilic chains involving two sulfonate (−SO_3_^–^) groups. In view of the emerging significance of development of new anticancer molecular strategies based in combination of Pt^2+^ complexes with human transferrin, we herein present studies of the interaction of a new cyclometalated Pt^2+^ with this protein.

The water-soluble 1.Cl was successfully synthesized by direct metalation of L^1^ (L^1^ = 3,3′-(1,3-phenylenebis­(1H-benzo­[d]­imidazole-2,1-diyl))­bis­(propane-1-sulfonate)) with K_2_[PtCl_4_] under a N_2_ atmosphere in acetic acid as shown in Scheme 1. The synthetic ligand L^1^ (N'^'C'^'N) and 1.Cl complex were pure according to elemental analysis (C, H, N, S), ^1^H, ^13^C NMR, IR-ATR, and mass spectrometry of ESI (Figures S2–S9).

**Scheme 1:**
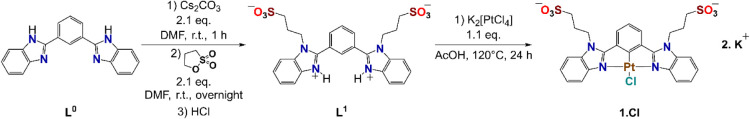
Chemical Synthesis of Anionic L^1^ ligand (N'^'C'^'N) and DPtCl1.Cl Complex

Efforts to obtain single crystals of the chloro complex 1.Cl were unsuccessful possibly due to repulsion between negatively charged aliphatic chains when the fragments [Pt­(N'^'C'^'N)­Cl] tend to stack. However, single crystals suitable of a related acetate derivative of 1.Cl (hereafter 1.Aco) obtained via 1.Cl in the presence of Mg­(AcO)_2_, were suitable for X-ray diffraction from a CH_3_OH-H_2_O solution (see Tables S1–S4 in the Supporting Information for crystallographic data, selected distances/angles around the Pt^2+^ atom, and hydrogen-bonding interactions within the crystal packing of this complex). Figure 1 depicts a perspective molecular view of the crystal of 1.Aco. X-ray structural analysis of 1.Aco shows that the unit cell contains two independent molecules of the solvated Pt^2+^ complex with the general formula [Pt­(N'^'C'^'N)­(AcO)]­(K^+^)_2_·CH_3_OH and [Pt­(N'^'C'^'N)­(AcO)]­Mg^2+.^CH_3_OH. The crystal structure of the acetate derivative (1.Aco) confirms a distorted square-planar Pt­(II) coordination geometry typical of cyclometalated N'^'C'^'N complexes (Figure S10). However, this structure does not directly represent the chloro complex 1.Cl in solution. The Aco organometallic structure is similar to that of the 1.Cl structure, which was not feasible to produce single crystals.

**Figure 1:**
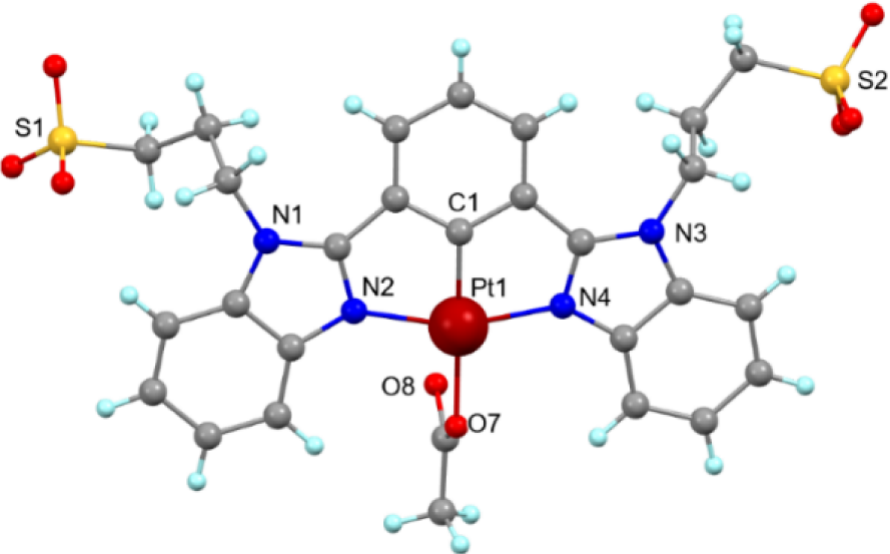
Perspective views of the anionic Pt^2+^-N'^'C'^'N complex, 1.Aco. For this complex, two independent molecules are observed in the unit cell (only one of which is shown). Counterions (K^+^, Mg^2+^) and additional solvent molecules (CH_3_OH, and H_2_O) are omitted for clarity.

In the crystal lattice, CH_3_OH and H_2_O molecules form multiple hydrogen-bonding interactions with sulfonate −SO_3_^–^ groups and solvate cations K^+^ and Mg^2+^.

To the best of our knowledge, this crystal is the first example of an anionic organometallic Pt^2+^-N̂ĈN complex of the type bearing two alkyl sulfonate (R-SO_3_^–^) chains.

As shown in Figure 2A, the electronic absorption spectrum of 1.Cl in pure water displayed intense band at 298 nm which is assigned as the intraligand (IL) [π–π*] transitions of the cyclometalated N'^'C'^'N ligand,^33^ and a lower-energy absorption band at 365 nm typically ascribed to a combination of IL transitions and metal-to-ligand charge transfer (MLCT), [d­(Pt) → π*­(N'^'C'^'N)] transitions.^37,40,46^ An aqueous solution of 1.Cl (5 mM, λ_ex_= 360 nm) at room temperature, well-resolved vibronic-structured emission bands at 498 nm, 535 nm, and 575 nm were observed with a lifetime of 1.64 μs (Figure 2B) and a quantum yield of Φ = 0.09.

**Figure 2:**
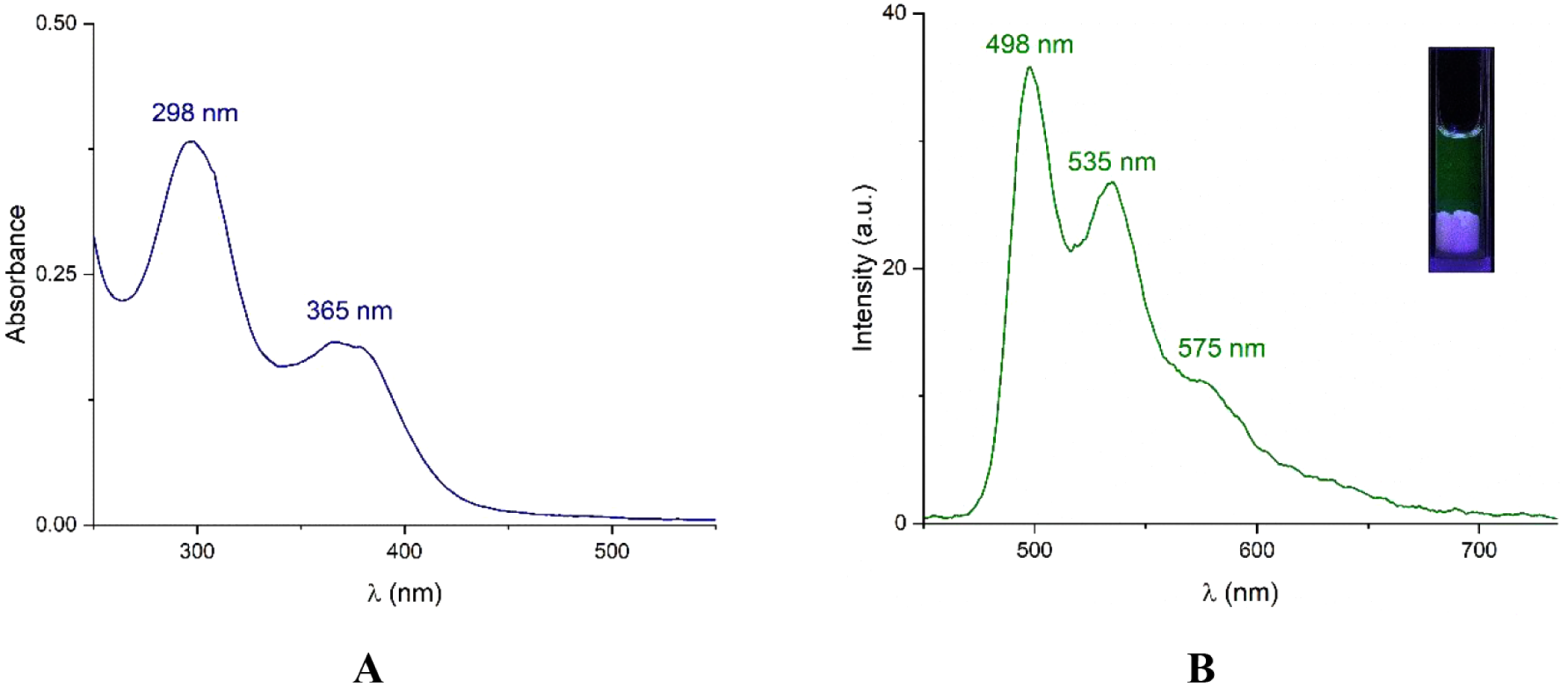
(A) UV–vis absorption and (B) emission spectra of 1.Cl (5 mM, λ_ex_= 360 nm) in water at 298 K. Inset: photograph of an aqueous solution of 1.Cl under UV light at 355 nm.

Water-soluble and hydrostable Pt^2+^ complexes are still rare in the literature and are highly desired for biological and biomedical applications. Valdes-García and coworkers reported a Pt-NCN complex bearing two tetraethylene glycol chains that exhibited stability in aqueous media and enabled the detection of GTP and CTP.^41,45^ In contrast, Baquero and coworkers reported a water-soluble Pt^2+^ complex incorporating a sulfonated dianionic N-heterocyclic carbene ligand; however, this system was tested as a catalyst for the hydration of alkynes rather than for biological applications.^42^ The incorporation of imidazole and pyrazol^46^ derivatives is a commonly found strategy in drug design, as exemplified by 1,3-bis­(1H-benzo­[d]­imidazol-2-yl)­benzene, which has demonstrated potential antitumoral activity.^16^ The incorporation of it as a ligand in a Pt^2+^ matrix, which mimics the cisplatin structure, could enhance the overall antitumoral activity by linking it to DNA in tumor cells through two chemical motifs. This idea motivated the synthesis of 1.Cl. After being determined in a cell viability test, 1.Cl achieves the cell growth inhibition of some cancerous cell lines. The inhibition rate reached over 50% (ratio of dead to total live cells in 1.Cl presence after 72 h of incubation) for colon (84.09%), breast (75.34%), leukemia (65.45%), and brain (52.26%) tumor cells. In contrast, inhibition ratios below 50% were found in the lung (37.55%) and prostate (23.84%) cells. In nontumorigenic COS-7 cells, the growth inhibition reaches 26.58%, a high level of inhibition. However, it is a vital observation, as COS-7 cell inhibition results in adverse effects on healthy tissues and therefore provides a basis for future improvements in the 1.Cl structure. Once the antitumoral activity of 1.Cl was quantified, the carrier systems, *a*Tf-1.Cl and *h*Tf-1.Cl, were observed in HEPES as a buffer under a 302 nm UV wavelength; the solutions exhibit fluorescence, 1.Cl was orange, *a*Tf-1.Cl bright yellow, and *h*Tf-1.Cl yellow, as seen in Figure 3. The displacement of orange to yellow, promoted by the presence of transferrin in *apo* and *holo* form, suggests interaction in solution; however, the brightness of *a*Tf-1.Cl compared with *h*Tf-1.Cl draws attention, and it may be the antenna effect. Even though this phenomenon is typically found in lanthanides (III), not in isolated platinum, the ligand L^1^ in 1.Cl could act as an antenna, absorbing and transferring energy to the platinum ion when it is coordinated, and more intensely, when 1.Cl interacts with transferrin protein, enhancing the total fluorescence, as suggested previously in platinum-aryl interactions.^47^ The implications of the change in symmetry from platinum­(II) square-planar to octahedral, such as those found in iridium­(III) or osmium­(II) symmetry, shall be studied in advance and could open the gate to using the complex *a*Tf-1.Cl as a photochemical sensor. The more emissive behavior of *a*Tf-1.Cl compared to *h*Tf-1.Cl could be attributed to the presence of iron ions (Fe^3+^) in *h*Tf, suggesting a competition between iron and platinum for coordination sites on the protein surface.

**Figure 3:**
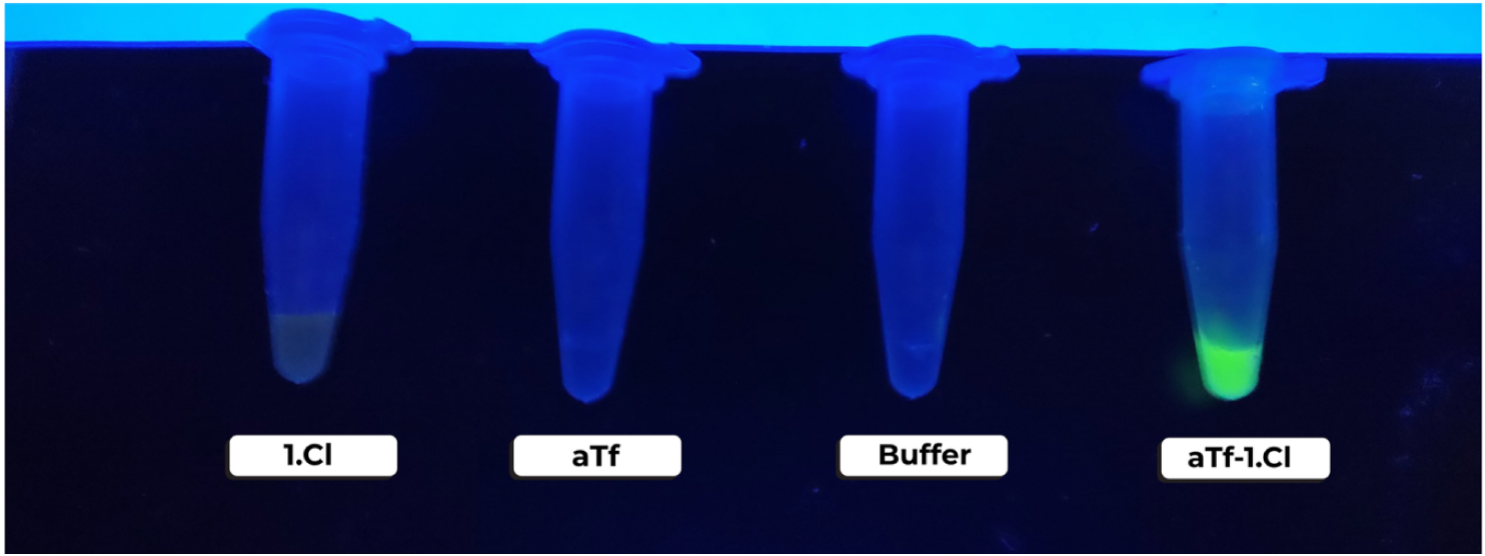
100 μL of fluorescent solution of the transferrin-1.Cl complex in HEPES buffer under a UV lamp (λ 302 nm), 1.0 mM of 1.Cl as a blank, and 1.0 mg·mL^–1^ protein solutions with the ligand 1.Cl. The tubes from left to right position are 1.Cl is the ligand in solution, *a*Tf stands for apo-Transferrin in buffer, and the third corresponds to the buffer only, while aTf-1.Cl is the protein and ligand in the buffer solution.

Taking into account the visual change in emission intensity between the free complex 1.Cl and 1.Cl in the presence of the *apo*- and *holo*-protein observed under UV light (*vide supra*), we investigated the affinity of complex 1.Cl toward transferrin (*a*Tf) through a titration experiment by steady-state fluorescence spectroscopy (Figure 4) in water at physiological pH (10 mM, HEPES, pH= 7.4). The addition of *a*Tf (0–9.3 mM) to a buffered aqueous solution of 1.Cl (5 mM, λ_ex_= 360 nm) strongly increases its green emission of all vibronic bands, *ca* 5-fold of initial intensity which is consistent with the brightness of the aqueous *a*Tf-1.Cl solution in Figure 3. The luminescent profile at 500 nm can be well fitted to a 1:1 binding isotherm by a nonlinear least-squares treatment using the eq 1)^41^ to output an apparent binding constant of *K*= (4.97 ± 0.12) × 10^6^ M^–1^. In eq 1, Δ*I*_*obs*_ corresponds to the observed change of fluorescent intensity, *I*_1.Cl_ is the intensity of the free Pt^2+^ complex, Δ*I*_∞_ corresponds to the change of intensity generated by the protein, [*a*Tf]_T_ and [1.Cl]_T_ are the total concentrations of the protein and Pt^2+^ ligand, respectively, and *K* is the *apparent* binding constant for a 1:1 stoichiometric model.

**Figure 4:**
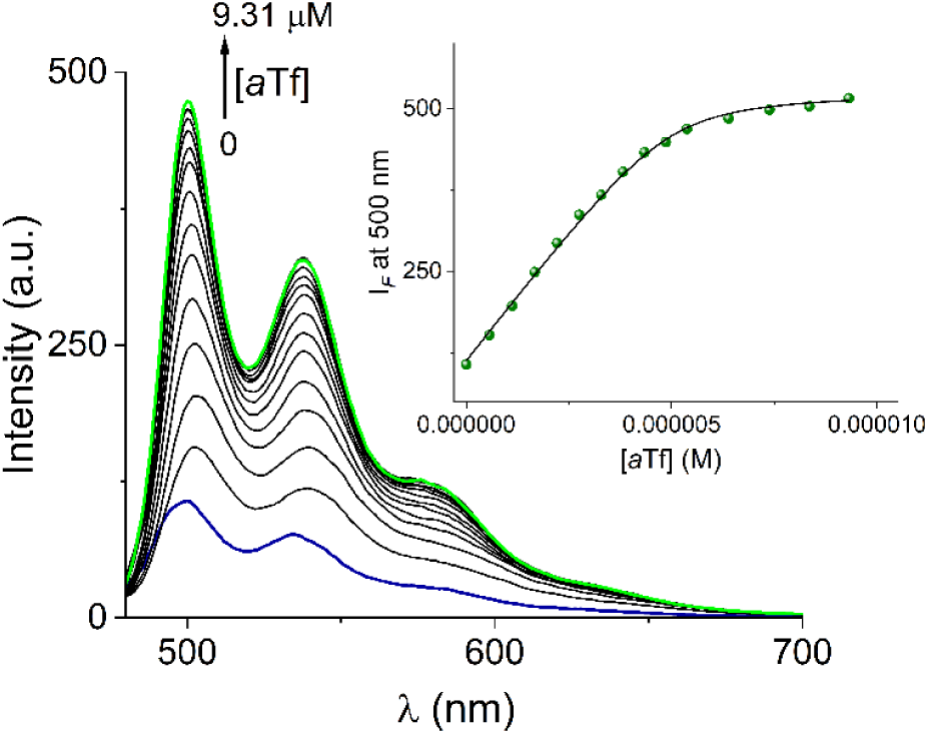
Changes of the fluorescent spectra of 1.Cl (λ_ex_ = 360 nm) upon the addition of *apo*-protein (0–9.31 μM). The inset shows the emission intensity at 500 nm as the *a*Tf concentration increases. The solid line was obtained by fitting the profile to a 1:1 stoichiometry.

Furthermore, the stoichiometry ratio of *a*Tf-1.Cl was verified by the continuous variation method (Job Plot, Figure S11), indicating a 1:1 Pt^2+^-ligand-protein supramolecular complex.

1 $$\Delta I_{obs}=I_{1.Cl}+0.5\Delta I_{\infty }\left\{{[aTf]}_{T}+{[1.Cl]}_{T}+\frac{1}{K}-{[{\left({\left[aTf\right]}_{T}+{\left[1.Cl\right]}_{T}+\frac{1}{K}\right)}^{2}-\left[4\right]{[1.Cl]}_{T}{[aTf]}_{T}]}^{0.5}\right\}$$

It was also observed the luminescence decay profile at 355 nm (Figure S12). Next, to investigate the energetic basis of the interaction between *a*TF and 1.Cl, titration of the *apo* protein with the synthetic ligand was performed by using isothermal titration calorimetry (ITC). The binding isotherm exhibited a well-defined sigmoidal shape, which was satisfactorily fitted by using a simple 1:1 binding model (Figure 5). The interaction was characterized by moderate affinity (*K*_*b*_ = 3.4 ± 0.5 × 10^6^ M^–1^), with a favorable enthalpic contribution (*ΔH*_*b*_ = −8.2 kcal·mol^–1^) and a marginally unfavorable entropic contribution (*TΔS*_*b*_ = −0.5 kcal·mol^–1^). In terms of elemental contributions, this thermodynamic signature indicates that the intrinsic contact interaction and associated conformational reorganization of the interacting molecules are more favorable than the enthalpic penalty associated with desolvation. Notably, the calorimetric isotherm displayed a clear inflection point and a well-defined saturation plateau, featuring characteristic of a dominant interacting event with a defined stoichiometry. Such behavior is difficult to reconcile with a purely nonspecific interaction involving multiple weakly interacting events, which typically yields shallow, poorly curved isotherms lacking a clear saturation regime. In this context, the near 1:1 stoichiometry suggested by the fitted parameter (η = 0.79 ± 0.01) should be interpreted cautiously, as deviations from unity are frequently observed in ITC experiments and often reflect the presence of a fraction of inactive or noninteracting protein, rather than true nonstoichiometric interacting mechanism.^48,49^ Accordingly, the stoichiometry parameter primarily acts as a corrective factor in the analysis. While contributions from weak, nonspecific interactions cannot be entirely excluded, discrimination between these scenarios ultimately requires complementary structural information.

**Figure 5:**
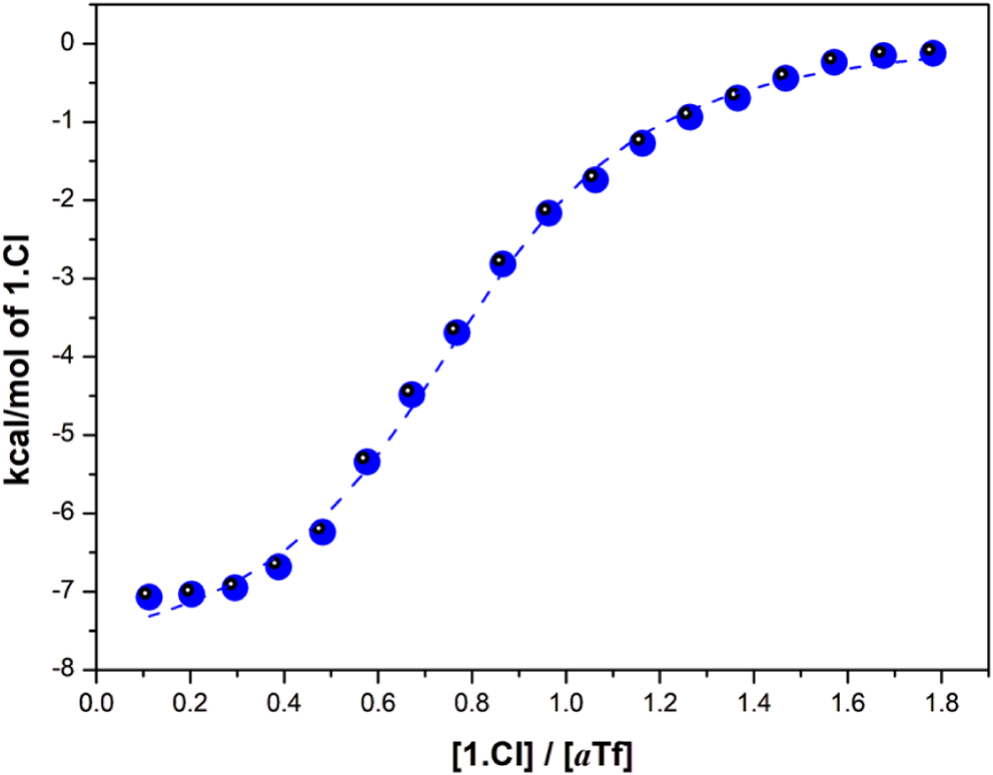
Thermodynamic characterization of the *a*Tf–1.Cl interaction by isothermal titration calorimetry. Calorimetric determination of the binding parameters between *a*TF and 1.Cl was by ITC. The isothermal binding curve corresponds to the titration of 45 μM *a*Tf with 0.4 mM 1.Cl. The dashed lines correspond to the best-fit model for a single, independent type of binding site. Measurement was performed at 303 K in 0.1 M HEPES, pH 7.4.

As an orthogonal approach to further explore the interaction, the effect of 1.Cl on the structural stability of *a*Tf was evaluated by differential scanning calorimetry (DSC). Although the two lobes of *a*Tf share relatively low sequence identity (∼44%), they exhibit a highly conserved bilobal architecture (RMSD ∼ 2.7 Å). Previous DSC studies have shown that the thermal unfolding of *a*Tf proceeds through two reversible transitions that can be described as weakly coupled two-state processes, with the C-terminal lobe exhibiting a melting temperature (Tm) ∼10 K lower than that of the N-terminal lobe.^50^ In agreement with these observations, the DSC endotherm obtained in this study for *a*Tf displayed two distinct peaks, which were satisfactorily fitted to a model of two independent unfolding transitions (Figure 6A). The lower-temperature peak, corresponding to the unfolding of the C-terminal lobe, yielded Tm_1_ = 334.1 ± 0.1 K and ΔH_1_ = 117.8 ± 0.6 kcal·mol^–1^, whereas the higher-temperature peak, corresponding to the N-terminal lobe, gave Tm_2_ = 343.9 ± 0.1 K and ΔH_2_ = 162.0 ± 0.5 kcal·mol^–1^.

**Figure 6:**
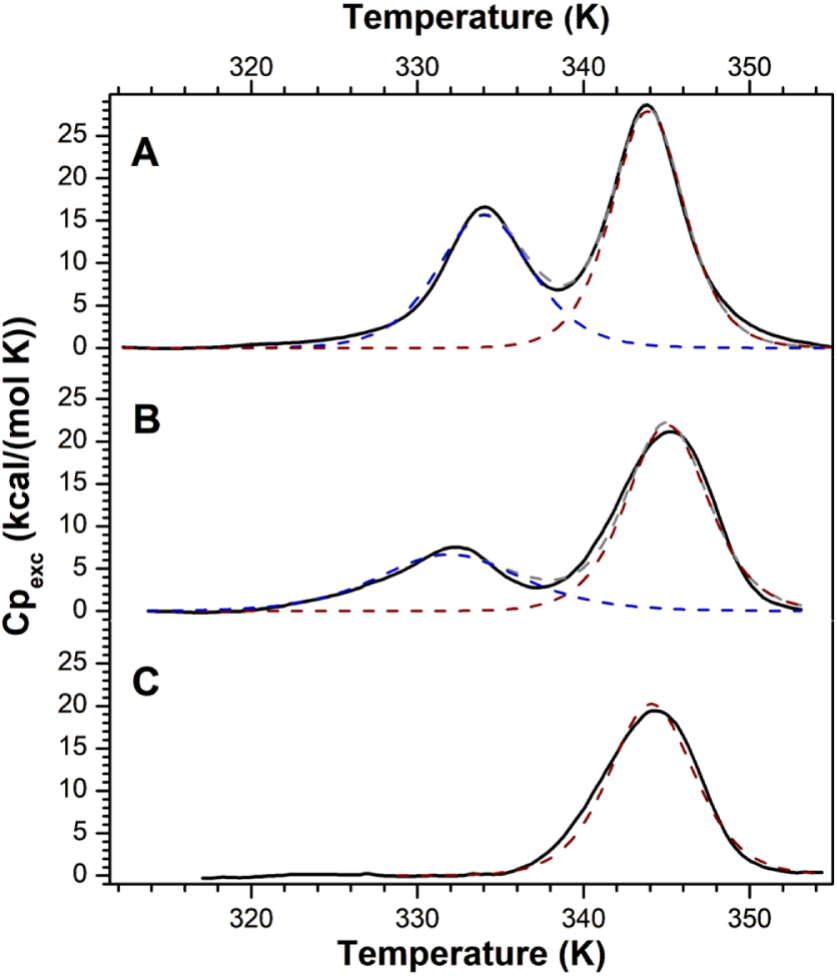
Effect of 1.Cl on the thermal stability of *a*Tf analyzed by differential scanning calorimetry. Thermal unfolding of 0.013 mM *a*Tf alone (A) and in the presence of 0.12 mM (B) and 0.25 mM 1.Cl (C), as determined by DSC. The experimental endotherms (solid lines) were fitted to an equilibrium model comprising two unfolding transitions. The dotted lines correspond to the global (gray) and individual transitions (blue and brown) resulting from the best fit of the model to the calorimetric traces. Measurements were performed in 0.1 M HEPES, pH 7.4, at a scan rate of 1 K·min^–1^.

Endotherms of *a*Tf were then recorded at increasing concentrations of 1.Cl (Figure 6B,C). The effect of the ligand on the two unfolding transitions was markedly distinct, primarily affecting the transition corresponding to the C-terminal lobe. At the lower ligand concentration, the first peak showed a slight decrease in Tm_1_ (∼ 275.15 K) together with a substantial reduction in the transition enthalpy (ΔH_1_ = 76.4 ± 0.9 kcal/mol), whereas the second peak remained essentially unchanged (Tm_2_ = 352.25 ± 0.1 K, ΔH_2_ = 144.2 ± 0.7 kcal/mol). At the higher 1.Cl concentration, the first transition disappeared entirely, while the second transition remained largely unaffected (Tm_2_ = 344.05 ± 0.1 K, ΔH_2_ = 138.0 ± 0.8 kcal/mol). Taken together, the ITC and DSC results are mutually consistent and support a preferential interaction between 1.Cl and *a*Tf that selectively perturbs the C-terminal lobe of the protein. Nevertheless, the definitive identification of a specific interaction site and the precise binding mode will require resolution of the three-dimensional structure of the complex.

### 3.2 Protein–Ligand Interaction in the Solid State

Once the relevant information on the interaction in solution was available, the system’s solid-state structures were obtained to identify its nature. The first attempt involved the batch crystallization of two components. The synthesis produced a micropowder unsuitable for diffraction. The strategy was then modified to employ cocrystallization methods,^51^ in which the first step was to obtain crystals of *a*Tf, as previously reported,^52^ followed by diffusing the 1.Cl solution into the crystal lattice. This process successfully employed vapor diffusion and counter diffusion in gel crystallization techniques,^53^ obtaining desirable crystals that could be diffused with 1.Cl. The platinum complex destroys the crystals, dissolving the *a*Tf crystals obtained by batch methods. Additionally, for capillary gels, crystal redissolution occurs at a slow to medium rate, resulting in a poor data set from synchrotron X-ray diffraction analysis. However, in the capillary crystallization system, recrystallization occurs via the Liesegang effect, in which small crystals form ring zones along the capillary. Pictures of the crystals of *a*Tf and 1.Cl are presented in Figure 7.

**Figure 7:**
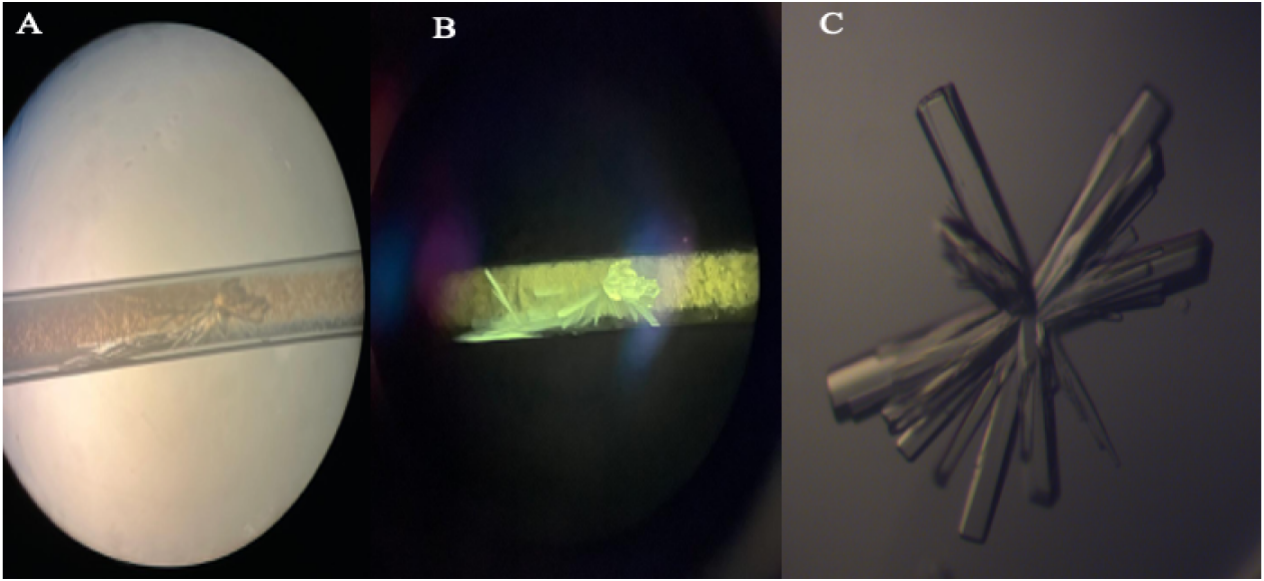
Crystals of (A) *a*Tf by capillary counter diffusion; (B) *a*Tf-1.Cl by the capillary counter-diffusion technique and (C) *a*Tf obtained by the vapor diffusion technique. Crystal growth began in the second week, but it is essential to keep it unperturbed at a crystallization temperature of 291 K. The fluorescent image (B) was irradiated at λ = 302 nm.

A total of 36 crystals were tested at the MANACÁ beamline, but most of them show very poor diffraction (>8 Å). Only one crystal diffracted at 3.2 Å, but the data were still of low quality (smeared reflections, low resolution, difficult indexing) (Figure 8). The cell parameters did not match those of the orthorhombic transferrin crystal structures reported in the PDB (7Q1L) but are closer to the open structure usually observed at lower pH.^52^ Additionally, the Wilson B-factor of the diffraction data is quite high, indicating crystalline disorder (Table 1). All attempts to optimize data processing and solve the structure by molecular replacement were unsuccessful (data not shown). However, using these X-ray data, we estimated that there are between 4 and 5 molecules in the asymmetric unit (ASU). For 4 molecules in the ASU, the estimated solvent content is 58.9%, and the Matthews coefficient is 2.99. On the other hand, for 5 molecules in the ASU, 48.6% solvent, and a Matthew coefficient of 2.39.

**Figure 8:**
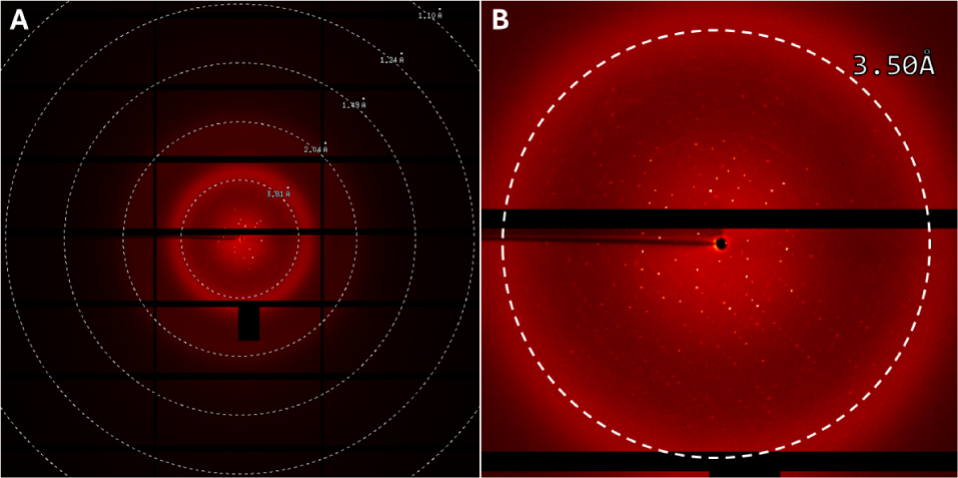
Diffraction pattern from the *a*Tf crystal: (A) wide view of the detector showing only low-resolution spots; (B) zoomed image showing weak spots around 3.5 Å resolution. This represents the best diffraction obtained from a total of 36 crystals tested.

The fluorescent activation by 1.Cl over *a*Tf supports the idea of a wide family of platinum-based agents with no oxo-coordination, distinct from carboplatin and oxoplatin. Additionally, 1.Cl is capable of being transported by transferrin and is easily detected due to its intrinsic fluorescence, allowing it to be identified in both its free and released forms. However, the iron influence on fluorescence, as shown in Figure 3, contrasts with the platinum enhancer effect and its relevance. Despite the fluorescence displayed by *a*Tf-1.Cl, we attempted to measure the association between *h*Tf and 1.Cl using ITC but were unable to do so, indicating significant interference between iron and platinum ions. This contrasts with the solid structure of the cisplatin *apo* and *holo*-transferrin complex,^14,15^ where no interference was identified, although the size of *a*Tf, which is around 100 times bigger than 1.Cl, the latter induces structural modifications in *a*Tf, modifies its bilobal glycoprotein intrinsic structure, as shown by the DSC curves of Figure 6, also, DSC curves illustrate the loss of structure of *a*Tf and a region highly thermally stable not influenced by 1.Cl, around 335–340 K.

The elucidation of the chemical nature of the interaction between 1.Cl and *a*Tf, the carrier transferrin, will be analyzed in the future. Additionally, the cytotoxic activity of 1.Cl should be optimized to improve its chemical structure, thereby reducing its cytotoxicity toward normal cells and increasing its selectivity toward tumor cells. Moreover, the fluorescent properties of system *a*Tf-1.Cl are being used to study the endocytosis process of 1.Cl by using flow cytometry. Solid-state structural elucidation is a preparatory step to delve deeper into the nature of the interaction and to gather additional information to improve the antitumoral system. The interaction between *h*Tf-1.Cl will be analyzed in the future to avoid potential competition at the binding sites between iron and platinum.

## 4 Conclusions

The complex *a*TF-1.Cl was synthesized, and the interaction protein ligand was measured in solution. The complex in solid form was also obtained, but the crystal did not meet the quality requirements for diffracting synchrotron radiation due to radiation damage. This could be attributed to the substantial change in osmotic pressure that occurs when the crystal is in contact with 1.Cl, as observed by the Liesegang effect over an *a*TF crystal. Even though we do not yet have the final chemical crystalline structure of the complex, the strategy of using carrier and platinum derivatives, as shown, is promising and warrants exploration with new variants of platinum-based compounds.

## Supplementary Material



## Notes

An FPLC-SEC chromatogram for apo-Transferrin (Figure S1). We have included Tables S1 to S5 for crystallographic data of 1.Aco (Table S1), selected bond distances (Å) and angles (°) around Pt atoms in 1.Aco (Table S2), as well as hydrogen bonds for 1.Aco [Å and °] (Table S3), for the atomic coordinates and equivalent isotropic displacement parameters (Table S4) and for the binding properties obtained from the calorimetric titration of 1.Cl over *a*Tf. Additionally, a ^1^H NMR (300 MHz, 298 K, DMSO-*d*_6_) spectrum of L^1^ was included (Figure S2), as well as ^13^C NMR (126 MHz, 25 °C, DMSO-*d*_6_) spectrum of L^1^ (Figure S3). An IR-ATR spectrum of L^1^ is shown (Figure S4). A positive scan MS-ESI spectrum of L^1^ in CH_3_OH (Figure S5), a ^1^H NMR (300 MHz, 298 K, DMSO-*d*_6_) spectrum of 1.Cl (Figure S6), a ^13^C NMR (76 MHz, 298 K, DMSO-*d*_6_) spectrum of 1.Cl (Figure S7), an IR-ATR spectrum of 1.Cl (Figure S8), and a negative scan MS-ESI spectrum of 1.Cl in CH_3_OH (Figure S9). Additionally, a perspective view of the unit cell of the 1.Cl crystal containing the two independent molecules and the counterions (Figure S10), a job continuous variation plot of the *a*Tf-1.Cl supramolecular complex system at a final concentration of 5 mM in buffered water (10 mM HEPES, pH= 7.4) (Figure S11). Finally, a luminescence decay profile (λ_ex_= 355 nm) of an aqueous solution of 1.Cl was also obtained (Figure S12) (PDF)

## References

[1] Arnesano, F. Platinum Complexes and Methionine Motif in Copper Transport Proteins, Interaction. In Encyclopedia of Metalloproteins.; Springer: New York, 2013.

[2] Arnesano F., Belviso B. D., Caliandro R., Falini G., Fermani S., Natile G., Siliqi D. (2011). Crystallographic
Analysis of Metal-Ion
Binding to Human Ubiquitin. Chem.-Eur. J..

[3] Fornaciari G. (2018). Histology
of ancient soft tissue tumors: A review. Int.
J. Paleopathol..

[4] Henderson R. H., French D., Stewart E., Smart D., Idica A., Redmond S., Eckstein M., Clark J., Sullivan R., Keeling P. (2023). Delivering the precision
oncology paradigm: reduced
R&D costs and greater return on investment through a companion
diagnostic informed precision oncology medicines approach. J. Pharm. Policy Pract..

[5] Rosenberg B., VanCamp L., Trosko J. E., Mansour V. H. (1969). Platinum compounds:
a new class of potent antitumour agents. Nature.

[6] Sahoo D., Deb P., Basu T., Bardhan S., Patra S., Sukul P. K. (2024). Advancements
in platinum-based anticancer drug development: A comprehensive review
of strategies, discoveries, and future perspectives. Bioorg. Med. Chem..

[7] Hambley T. W. (2001). Platinum
binding to DNA: Structural controls and consequences. J. Chem. Soc., Dalton Trans..

[8] Florea A. M., Büsselberg D. (2011). Cisplatin
as an anti-tumor drug: cellular mechanisms
of activity, drug resistance and induced side effects. Cancers.

[9] Chen Y., Wang J., Wang J., Wang L., Tan X., Tu K., Tong X., Qi L. (2016). Aptamer Functionalized Cisplatin-Albumin
Nanoparticles for Targeted Delivery to Epidermal Growth Factor Receptor
Positive Cervical Cancer. J. Biomed. Nanotechnol..

[10] Yin W.-J., Huang Y.-J., Zhu Q., Lin X.-Q., Piao H.-L., Yu Q.-Q., Lai C.-H., Zhou G.-L., Zhou L.-Y., Liu K. (2024). Hypoalbuminemia
and cisplatin-induced acute kidney
injury. Front. Pharmacol..

[11] Luo L. Z., Jin H. W., Huang H. Q. (2012). Transferrin-cisplatin specifically
deliver cisplatin to HepG2 cells in vitro and enhance cisplatin cytotoxicity. J. Proteomics.

[12] Lin X., Okuda T., Holzer A., Howell S. B. (2002). The copper transporter
CTR1 regulates cisplatin uptake in Saccharomyces cerevisiae. Mol. Pharmacol..

[13] Niitsu Y., Kohgo Y., Nishisato T., Kondo H., Kato J., Urushizaki Y., Urushizaki I. (1987). Transferrin receptors in human cancerous
tissues. Tohoku J. Exp. Med..

[14] Troisi R., Galardo F., Ferraro G., Lucignano R., Picone D., Marano A., Trifuoggi M., Sica F., Merlino A. (2025). Cisplatin/Apo-Transferrin Adduct:
X-ray Structure and Binding to the Transferrin Receptor 1. Inorg. Chem..

[15] Troisi R., Galardo F., Ferraro G., Sica F., Merlino A. (2023). Cisplatin
Binding to Human Serum Transferrin: A Crystallographic Study. Inorg. Chem..

[16] Valdes-García J., Viviano-Posadas A. O., Rivera-Chávez J., Ramírez-Apan T., Martínez-Vargas S., Aguirre-Hernández E., German-Acacio J. M., Morales-Morales D., Dorazco-González A. (2022). Crystal structures
and study of interaction mode of bis-benzimidazole-benzene derivatives
with DNA. J. Mol. Struct..

[17] Yin S. Y., Sun S. S., Pan M., Fan Y. Z., Chen Y. X., Wang H. P., Fan Y. N. (2017). Water soluble
Ir­(III) complexes from
sulfonate-modified cyclometalating ligand. Inorg.
Chem. Commun..

[18] Bruker AXS Inc. APEX 2 Software Suite; Bruker AXS Inc.: Madison, Wisconsin, USA, 2004.

[19] Bruker AXS Inc. Bruker SAINT And SADABS; Bruker AXS Inc.: Madison, Wisconsin, USA, 2007.

[20] Sheldrick G. M. (2008). A short
history of SHELX. Int. Union Crystallogr., Crystallogr..

[21] Hübschle C. B., Sheldrick G. M., Dittrich B. (2011). ShelXle: a Qt graphical user interface
for SHELXL. J. Appl. Crystallogr..

[22] Vichai V., Kirtikara K. (2006). Sulforhodamine
B colorimetric assay for cytotoxicity
screening. Nat. Protoc..

[23] Skehan P., Storeng R., Scudiero D., Monks A., McMahon J., Vistica D., Warren J. T., Bokesch H., Kenney S., Boyd M. R. (1990). New colorimetric
cytotoxicity assay for anticancer-drug
screening. J. Natl. Cancer Inst..

[24] Kabsch W. (2010). XDS. Acta Crystallogr..

[25] Gómez-Velasco H., Rojo-Domínguez A., García-Hernández E. (2020). Enthalpically-driven
ligand recognition and cavity solvation of bovine odorant binding
protein. Biophys. Chem..

[26] Labra-Núñez A., Cofas-Vargas L. F., Gutiérrez-Magdaleno G., Gómez-Velasco H., Rodríguez-Hernández A., Rodríguez-Romero A., García-Hernández E. (2021). Energetic and structural effects
of the Tanford transition on ligand recognition of bovine β-lactoglobulin. Arch. Biochem. Biophys..

[27] Tam A. Y. Y., Tsang P. K., Chan M. Y., Zhu N., Yam V. W. W. (2011). A luminescent
cyclometalated platinum­(II) complex and its green organic light emitting
device with high device performance. Chem. Commun..

[28] Wang Z., Sun Z., Hao X. Q., Niu J. L., Wei D., Tu T., Gong J. F., Song M. P. (2014). Neutral and cationic NCN pincer platinum­(II)
complexes with 1,3-bis­(benzimidazol-2′-yl)­benzene ligands:
Synthesis, structures, and their photophysical properties. Organometallics.

[29] Wang Z., Niu J. L., Zhang L. Z., Guo J. W., Hao X. Q., Song M. P. (2014). Synthesis, characterization
and photophysical properties
of the pincer platinum­(II) complexes with m-bis­(benzimidazol-2′-yl)­benzene
ligand. Tetrahedron.

[30] Qiu Y., Feng Y., Zhao Q., Wang H., Guo Y., Qiu D. (2020). White light emission
from a green cyclometalated platinum­(ii) terpyridylphenylacetylide
upon titration with Zn­(ii) and Eu­(iii). Dalton
Trans..

[31] Chan M. H., Wong H. L., Yam V. W. (2016). Synthesis and Photochromic Studies
of Dithienylethene-Containing Cyclometalated Alkynylplatinum­(II) 1,3-Bis­(N-alkylbenzimidazol-2’-yl)­benzene
Complexes. Inorg. Chem..

[32] Farley S. J., Rochester D. L., Thompson A. L., Howard J. A., Williams J. A. (2005). Controlling
emission energy, self-quenching, and excimer formation in highly luminescent
N∧C∧N-coordinated platinum­(II) complexes. Inorg. Chem..

[33] Albrecht M., van Koten G. (2001). Platinum Group
Organometallics Based on “Pincer”
Complexes: Sensors, Switches, and Catalysts. Angew. Chem., Int. Ed..

[34] Chan A. K.-W., Lam E. S.-H., Tam A. Y.-Y., Tsang D. P.-K., Lam W. H., Chan M.-Y., Wong W.-T., Yam V. W.-W. (2013). Synthesis and
characterization of luminescent cyclometalated platinum­(II) complexes
of 1,3-bis-hetero-azolylbenzenes with tunable color for applications
in organic light-emitting devices through extension of π conjugation
by variation of the heteroatom. Chem.-Eur. J..

[35] Chan A. K., Ng M., Wong Y. C., Chan M. Y., Wong W. T., Yam V. W. (2017). Synthesis
and Characterization of Luminescent Cyclometalated Platinum­(II) Complexes
with Tunable Emissive Colors and Studies of Their Application in Organic
Memories and Organic Light-Emitting Devices. J. Am. Chem. Soc..

[36] Lam E. S., Tsang D. P., Lam W. H., Tam A. Y., Chan M. Y., Wong W. T., Yam V. W. (2013). Luminescent
platinum­(II) complexes
of 1,3-bis­(N-alkylbenzimidazol- 2’-yl)­benzene-type ligands
with potential applications in efficient organic light-emitting diodes. Chemistry.

[37] Dorazco-Gonzalez A. (2014). Chemosensing
of chloride based on a luminescent platinum­(II) NCN pincer complex
in aqueous media. Organometallics.

[38] Dorazco-González, A. Chapter 27-Use of Pincer Compounds as Metal-Based Receptors for Chemosensing of Relevant Analytes Pincer Compounds: Chemistry And Applications Elsevier 2018 587–597 10.1016/B978-0-12-812931-9.00027-X

[39] Hao X.-Q., Xu Y.-X., Yang M.-J., Wang L., Niu J.-L., Gong J.-F., Song M.-P. (2012). A cationic
NCN pincer platinum­(II)
aquo complex with a bis­(imidazolinyl) phenyl ligand: Studies toward
its synthesis and asymmetric Friedel-Crafts alkylation of indoles
with nitroalkenes. Organometallics.

[40] Zhao W. W., Zhao J., Guo H., Sun J., Ji S., Wang Z. (2012). Long-lived room-temperature near-IR
phosphorescence of BODIPY in
a visible-light-harvesting N'^'C'^'N
PtII-acetylide complex
with a directly metalated BODIPY chromophore. Chem.-Eur. J..

[41] Valdes-García J., Zamora-Moreno J., Pinzón-Vanegas C., Viviano-Posadas A. O., Martínez-Otero D., Barroso-Flores J., Ortiz-Lopez B., Ortiz-Navarrete V. F., Dorazco-González A. (2023). Selective
Luminescent Chemosensing of Chloride Based on a Cyclometalated Platinum­(II)
Complex in Water: Crystal Structures, Spectroscopic Studies, Extraction,
and Bioimaging. Inorg. Chem..

[42] Baquero E. A., Silvestri G. F., Gómez-Sal P., Flores J. C., de Jesús E. (2013). Sulfonated
Water-Soluble N-Heterocyclic Carbene Silver­(I) Complexes: Behavior
in Aqueous Medium and as NHC-Transfer Agents to Platinum­(II). Organometallics.

[43] Almássy A., Nagy C. E., Bényei A. C., Joó F. (2010). Novel Sulfonated
N-Heterocyclic Carbene Gold­(I) Complexes: Homogeneous Gold Catalysis
for the Hydration of Terminal Alkynes in Aqueous Media. Organometallics.

[44] Solomatina A. I., Chelushkin P. S., Krupenya D. V., Podkorytov I. S., Artamonova T. O., Sizov V. V., Melnikov A. S., Gurzhiy V. V., Koshel E. I., Shcheslavskiy V. I., Tunik S. P. (2017). Coordination to
Imidazole Ring Switches on Phosphorescence of Platinum Cyclometalated
Complexes: The Route to Selective Labeling of Peptides and Proteins
via Histidine Residues. Bioconjugate Chem..

[45] Valdes-García J., Vonlanthen M., Cuétara-Guadarrama F., Garcia-Bassoco D., Valdés-Negrín H. L., López-Pérez A., Rivera E., Hernández-Ortega S., Dorazco-González A. (2026). A Dual Luminescent
and Chromogenic Pt-NCN Complex for the Detection of GTP and CTP in
Aqueous Media. Inorg. Chem..

[46] Abdelrahman A. M., Fahmi A. A., Rizk S. A., El-Helw E. A. (2023). Synthesis, DFT and
Antitumor Activity Screening of Some New Heterocycles Derived from
2,2’-(2-(1,3-Diphenyl-1H-Pyrazol-4-yl)­Ethene-1,1-Diyl)­Bis­(4H-Benzo­[d]­[1,3]­Oxazin-4-One). Polycyclic Aromat. Compd.

[47] Herberger J., Winter R. F. (2019). Platinum emitters
with dye-based σ-aryl ligands. Coord.
Chem. Rev..

[48] Luviano A., Cruz-Castañeda R., Sánchez-Puig N., García-Hernández E. (2019). Cooperative
energetic effects elicited
by the yeast Shwachman-Diamond syndrome protein (Sdo1) and guanine
nucleotides modulate the complex conformational landscape of the elongation
factor-like 1 (Efl1) GTPase. Biophys. Chem..

[49] Bastos M., Abian O., Johnson C. M., Ferreira-da-Silva F., Vega S., Jimenez-Alesanco A., Ortega-Alarcon D., Velazquez-Campoy A. (2023). Isothermal titration
calorimetry. Nat. Rev. Methods Primers.

[50] Lin L. N., Mason A. B., Woodworth R. C., Brandts J. F. (1994). Calorimetric studies
of serum transferrin and ovotransferrin. Estimates of domain interactions,
and study of the kinetic complexities of ferric ion binding. Biochemistry.

[51] Gutiérrez-Quezada, A. E. ; Arreguín-Espinosa, R. ; Moreno, A. Protein Crystal Growth Methods. In Springer Handbook of Crystal Growth, Dhanaraj, G. ; Byrappa, K. ; Prasad, V. ; Dudley, M. , Eds.; Springer: Berlin, Heidelberg, 2010; pp. 1583–1605.

[52] Campos-Escamilla C., Siliqi D., Gonzalez-Ramirez L. A., Lopez-Sanchez C., Gavira J. A., Moreno A. (2021). X-ray Characterization of Conformational
Changes of Human Apo- and Holo-Transferrin. Int. J. Mol. Sci..

[53] Moreno, A. Advanced Methods of Protein Crystallization In Protein Crystallography: methods and Protocols, Wlodawer, A. ; Dauter, Z. ; Jaskolski, M. Eds.; Springer: New York, NY, 2017, pp. 51–76. DOI: 10.1007/978-1-4939-7000-1_3 28573569

