# Supplementary material for: A Chemical Approach to the Synthesis of an Antitumoral Pt-Based Drug Molecule Using a Protein-Ligand Interaction as a Carrier System
Source: ACS Omega. 2026 Apr 22;11(17):25515–25. doi: 10.1021/acsomega.5c13604 (PMC13150575; doi:10.1021/acsomega.5c13604)
Supplement: Supplementary file 1 [file ao5c13604_si_001.pdf]

A chemical approach to the synthesis of an antitumoral molecule of Pt-based drug using the interaction's strategy protein-ligand as a carrier system

*Ulises Galindo-García[1], Rodrigo Flores-Manzo[1], Monserrat Yesenia Garrido-Santos[1], Alan Juárez-Barragán[1], Josue Valdés-García[2], Diego Martínez-Otero[3], Andrey Fabricio Ziem Nascimento[4], Mayra Cuéllar-Cruz[5], Enrique García-Hernandez[1], Alejandro Dorazco-González[1] and Abel Moreno[1]**

[1]Instituto de Química, Universidad Nacional Autónoma de México. Avenida Universidad 3000. Colonia UNAM. Mexico City 04510. Mexico.

[2]Instituto de Investigaciones en Materiales, Universidad Nacional Autónoma de México. Avenida Universidad 3000. Colonia UNAM. Mexico City 04510. Mexico.

[3]Center for Research in Sustainable Chemistry (CCIQS) UAEMéx-UNAM.

[4]Brazilian Synchrotron Light Laboratory (LNLS), Brazilian Center for Research in Energy and Materials (CNPEM), Zip Code 13083-970, Campinas, Sao Paulo, Brazil.

[5]Departamento de Biología, División de Ciencias Naturales y Exactas, Campus Guanajuato, Universidad de Guanajuato, Noria Alta S/N, Col. Noria Alta, 36050, Guanajuato, Guanajuato, México

Supporting Information

1. Size-exclusion Chromatography of *a*Tf

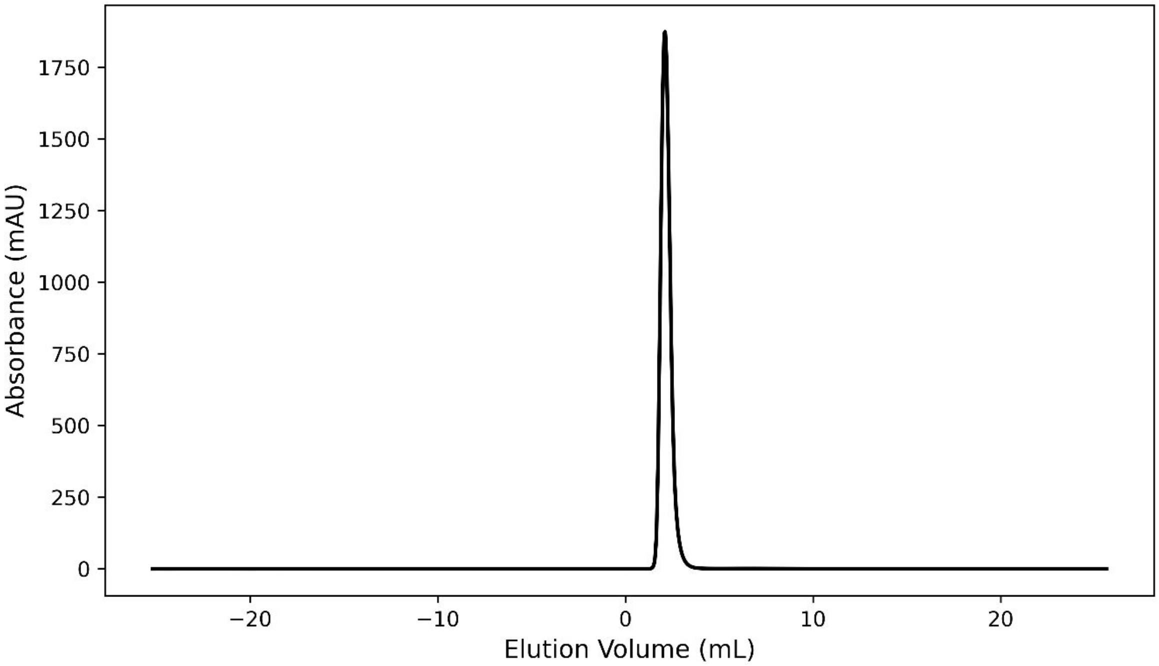

Figure S1. FPLC-SEC chromatogram *a*Tf

2. Crystal structure and selected geometric parameters of 1.Aco

Table S1. Crystallographic data for 1.Aco.

	**1.Aco**
Empirical formula	$C_{62}H_{80}K_2MgN_8O_{24}Pt_2S_4$
Mol. Weight / g mol^{-1}	1942.27
Crystal system	Monoclinic
Space group	$P2_1/c$
a / Å	15.2598(19)
b / Å	16.543(2)
c / Å	14.8467(19)
α / °	90°
β / °	94.678(2)°
γ / °	90°
Volume / Å^3	3735.4(8)
Z	2
Density / mg cm^{-3}	1.727
Crystal size	0.492 x 0.370 x 0.283 mm3
Temperature / K	100(2)
Abs. Coeff. / mm^{-1}	4.053
Θ range / °	2.214 to 27.446
Index ranges	-19<=h<=19, -21<=k<=21, -19<=l<=19
Reflections collected	80220
Max. & min. transmission	0.7456 and 0.5959
Data / restraints / param.	8530 / 156 / 538
Goodness-of-fit on F^2	1.045
Final *R* indices [I > 2 s (I)]	R1 = 0.0299, wR2 = 0.0758
R indices (all data)	R1 = 0.0309, wR2 = 0.0765
Larg. Diff. peak and hole / eÅ^{-3}	2.985 and -0.908

Table S2. Atomic coordinates (x 104) and equivalent isotropic displacement parameters (Å2x 103) for 1.Aco. U(eq) is defined as one third of the trace of the orthogonalized Uij tensor.

	x	y	z	U(eq)
Pt(1)	5142(1)	5300(1)	6060(1)	18(1)
S(1)	6963(1)	10007(1)	5672(1)	26(1)
S(2)	-296(1)	4260(1)	6537(1)	47(1)
O(1)	7255(2)	9635(2)	4850(2)	34(1)
O(9)	9408(3)	9798(4)	6166(4)	91(2)
O(10)	10461(7)	8887(5)	5019(7)	91(2)
C(29)	10898(8)	8354(8)	5735(9)	104(4)
O(10A)	10452(19)	8562(13)	4880(20)	108(4)
C(29A)	10709(18)	7712(12)	5130(20)	97(4)
O(11)	8857(3)	9682(4)	4267(4)	70(2)
C(30)	8713(6)	9160(7)	3494(5)	87(3)
O(11A)	9030(30)	9570(30)	3900(30)	82(4)
C(30A)	9270(30)	8720(30)	3830(40)	80(4)
N(1)	6012(2)	7522(2)	5303(2)	21(1)
C(1)	4254(2)	6040(2)	5536(2)	19(1)
N(2)	5906(2)	6263(2)	5817(2)	21(1)
N(3)	2596(2)	4497(2)	5938(2)	23(1)
N(4)	4053(2)	4624(2)	6152(2)	21(1)
C(31)	6240(4)	11742(3)	7372(3)	51(1)
K(1)	-1216(9)	2149(7)	7046(8)	166(4)
K(1A)	-696(11)	2278(8)	6112(11)	138(4)
K(1B)	-579(19)	2288(18)	7780(20)	249(9)
K(1C)	-336(14)	2062(12)	6785(16)	191(6)
C(2)	4504(2)	6820(2)	5288(2)	20(1)
O(2)	6354(2)	10668(2)	5447(2)	38(1)

O(3)	7694(2)	10233(2)	6316(2)	37(1)
O(4)	-1201(3)	4490(3)	6544(4)	69(1)
O(5)	-167(3)	3787(3)	5731(4)	78(1)
O(6)	94(3)	3869(3)	7343(3)	76(1)
O(7)	6100(2)	4524(2)	6703(2)	39(1)
O(8)	6044(3)	5083(2)	8117(2)	51(1)
C(3)	3855(2)	7371(2)	4974(2)	24(1)
C(4)	2979(3)	7120(2)	4887(3)	27(1)
C(5)	2721(2)	6345(2)	5127(2)	25(1)
C(6)	3370(2)	5802(2)	5472(2)	20(1)
C(7)	5457(2)	6906(2)	5453(2)	20(1)
C(8)	6859(2)	7269(2)	5578(2)	23(1)
C(9)	7664(3)	7663(3)	5567(3)	29(1)
C(10)	8397(3)	7241(3)	5904(3)	36(1)
C(11)	8329(3)	6447(3)	6228(3)	35(1)
C(12)	7536(3)	6050(2)	6230(3)	28(1)
C(13)	6788(2)	6479(2)	5901(2)	23(1)
C(14)	5783(2)	8318(2)	4912(2)	23(1)
C(15)	5563(2)	8934(2)	5628(2)	22(1)
C(16)	6350(2)	9263(2)	6224(2)	22(1)
C(17)	3302(2)	4981(2)	5835(2)	21(1)
C(18)	2915(3)	3796(2)	6363(2)	24(1)
C(19)	2474(3)	3117(2)	6661(3)	30(1)
C(20)	2998(3)	2515(2)	7064(3)	33(1)
C(21)	3913(3)	2575(2)	7170(2)	30(1)
C(22)	4352(3)	3252(2)	6883(2)	26(1)
C(23)	3830(3)	3872(2)	6480(2)	23(1)
C(24)	1663(3)	4668(2)	5738(3)	27(1)
C(25)	1306(3)	5082(3)	6546(3)	32(1)
C(26)	312(3)	5177(3)	6445(3)	37(1)
C(27)	6380(3)	4548(2)	7551(3)	28(1)
C(28)	7093(3)	3992(3)	7940(3)	42(1)
Mg(1)	10000	10000	5000	46(1)
O(12)	5576(2)	11238(2)	6893(3)	52(1)

Table S3. Selected bond distances (Å) and angles (°) around Pt atoms in 1.Aco.

**1.Aco**	
Pt(1)-C(1)	1.941(3)
Pt(1)-N(4)	2.018(3)
Pt(1)-N(2)	2.023(3)
Pt(1)-O(7)	2.114(3)
C(1)-Pt(1)-N(4)	79.88(13)
C(1)-Pt(1)-N(2)	79.99(13)
N(4)-Pt(1)-N(2)	159.78(12)
C(1)-Pt(1)-O(7)	176.69(12)
N(4)-Pt(1)-O(7)	100.10(13)
N(2)-Pt(1)-O(7)	99.88(13)

Table S4. Hydrogen bonds for 1.Aco [Å and °].

D-H…A	d(D-H)	d(H…A)	d(D…A)	<(DHA)
O(9)-H(9A)…S(1)	0.847(10)	3.01(5)	3.760(5)	148(7)
O(9)-H(9A)…O(11^a)	0.847(10)	2.44(7)	2.883(8)	113(6)
O(9)-H(9A)…O(3)	0.847(10)	2.11(6)	2.740(6)	131(7)
O(9)-H(9B)…O(10^a)	0.851(10)	2.35(7)	2.863(11)	119(6)
O(10^a)-H(10A^a)…O(9)#3	0.84	2.26	2.816(13)	124.1
O(11^a)-H(11A^a)…S(1)	0.84	2.96	3.739(6)	155.3
O(11^a)-H(11A^a)…O(1)	0.84	1.91	2.660(6)	147.5
O(11A^b)-H(11B^b)…O(1)	0.84	2.36	3.15(5)	158.3
O(12)-H(12A)…S(1)	0.84	2.86	3.542(4)	139.4
O(12)-H(12A)…O(2)	0.84	1.88	2.706(5)	165.8

Symmetry transformations used to generate equivalent atoms:
#1 -x+1,-y+1,-z+1 #2 x-1,y-1,z #3 -x+2,-y+2,-z+1
#4 x+1,y+1,z

3. Characterization of L^1 and 1.Cl

3.1 Characterization of 3,3'-(1,3-phenylenebis(1H-benzo[d]imidazole-3-ium-2,1-diyl))bis(propane-1-sulfonate), L^1

3.1.1 ^{1}H and ^{13}C, Nuclear Magnetic Resonance of L^1

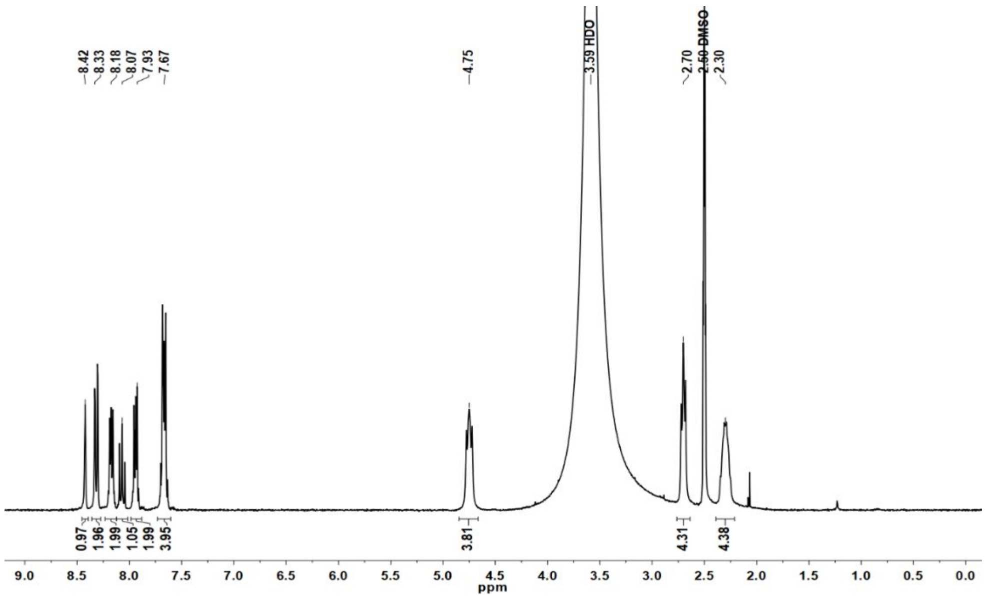

Figure S2. ^{1}H NMR (300 MHz, 298 K, DMSO-d_6) spectrum of L^1.

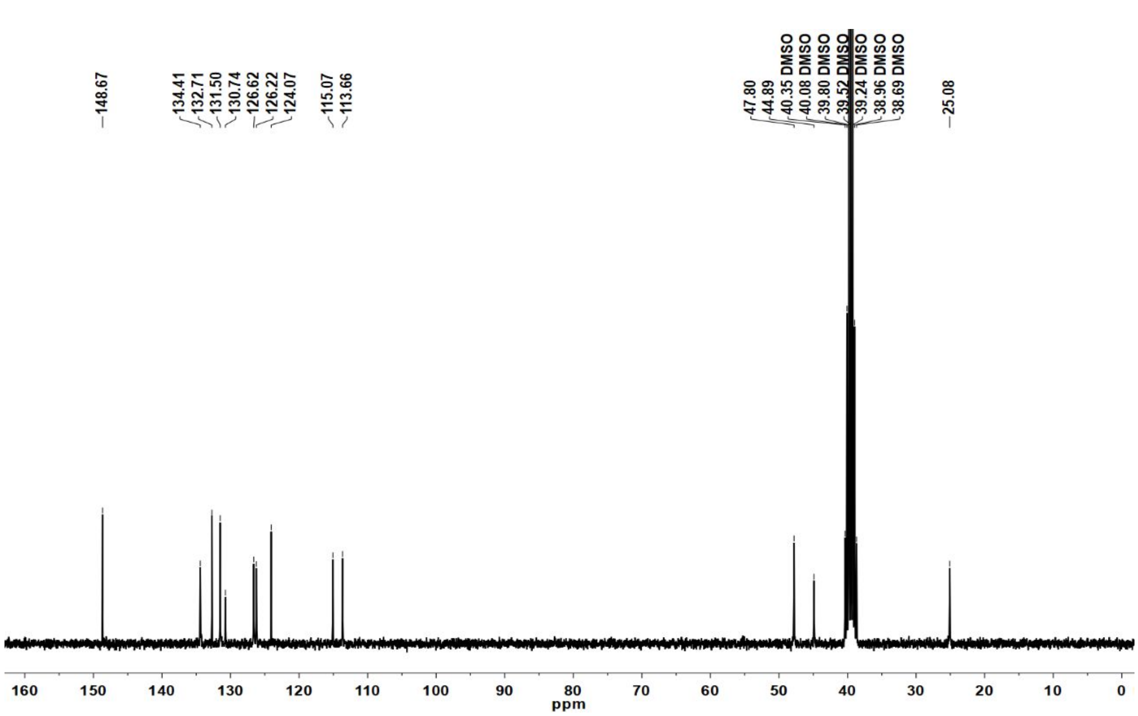

Figure S3. ^{13}C NMR (126 MHz, 298 K, DMSO-d_6) spectrum of L^1.

3.1.2 Infrared - Attenuated total reflection IR-ATR of L^1

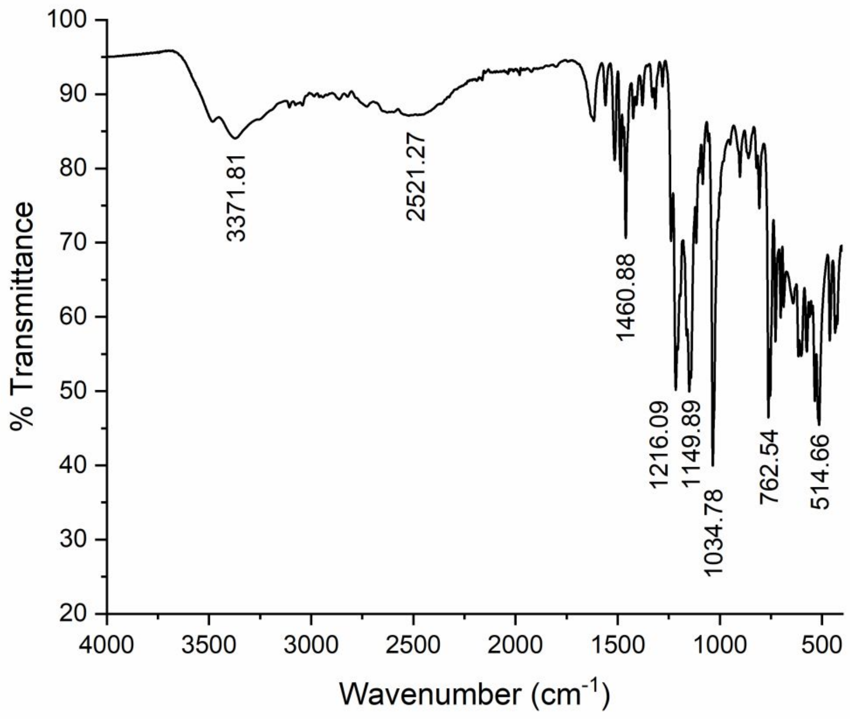

Figure S4. IR-ATR spectrum of L^1.

3.1.3 Electrospray Ionization Mass Spectroscopy ESI-MS of L^1

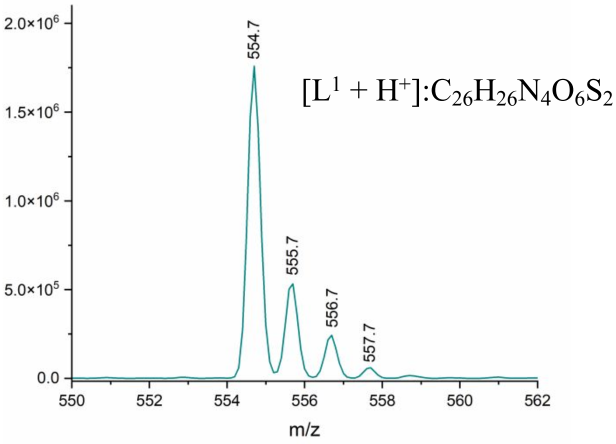

Theoretical isotope distribution

Figure S5. Positive scan MS-ESI spectrum of $\mathbf{L^1}$ in CH_3OH.

3.2 Characterization of 1.Cl

3.2.1 ¹H and ¹³C, Nuclear Magnetic Resonance of 1.Cl

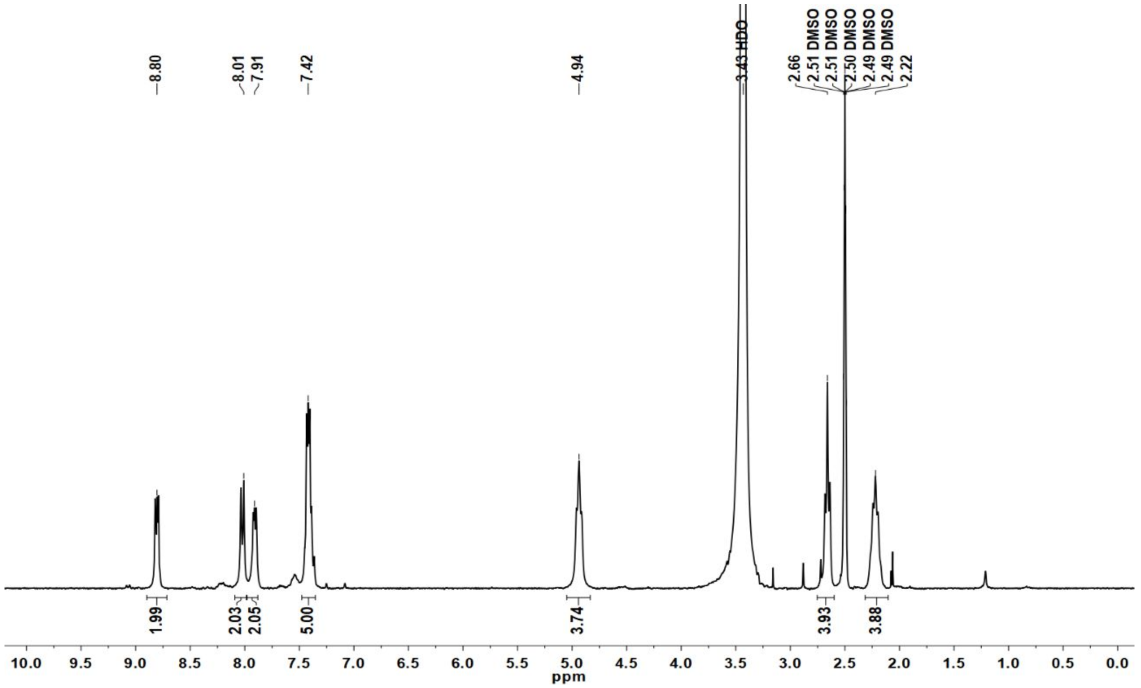

Figure S6. ^{1}H NMR (300 MHz, 298 K, DMSO-d_6) spectrum of 1.Cl.

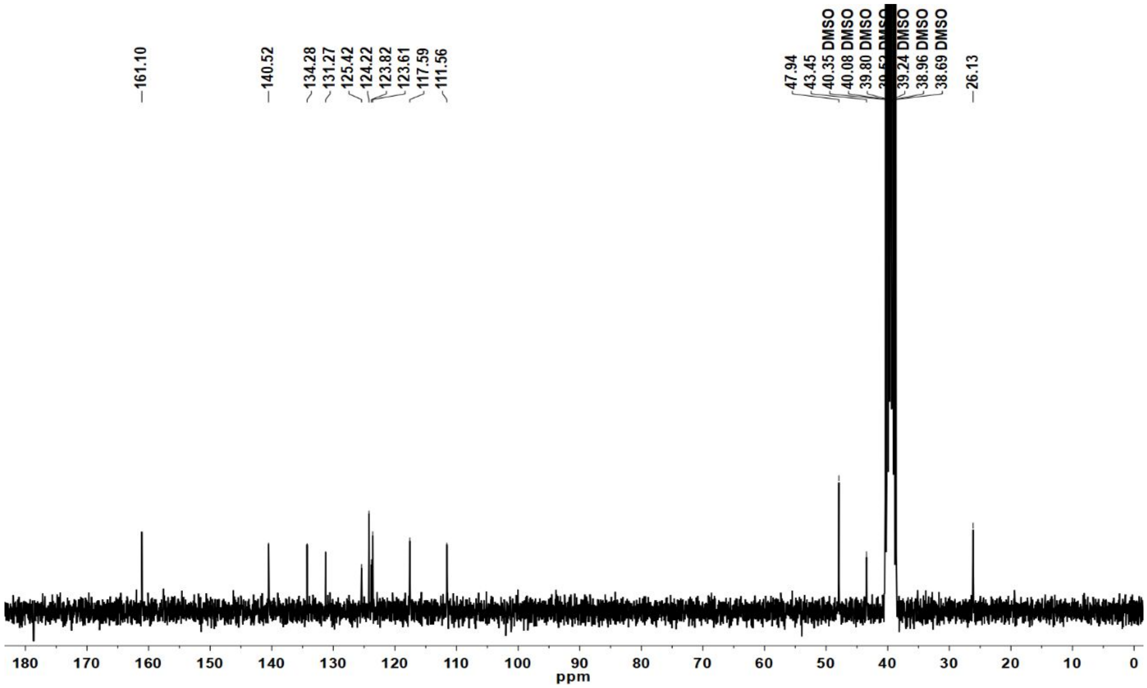

Figure S7. ^{13}C NMR (76 MHz, 298 K, DMSO-d_6) spectrum of 1.Cl.

3.2.2 Infrared - Attenuated total reflection IR-ATR of ***1.Cl***

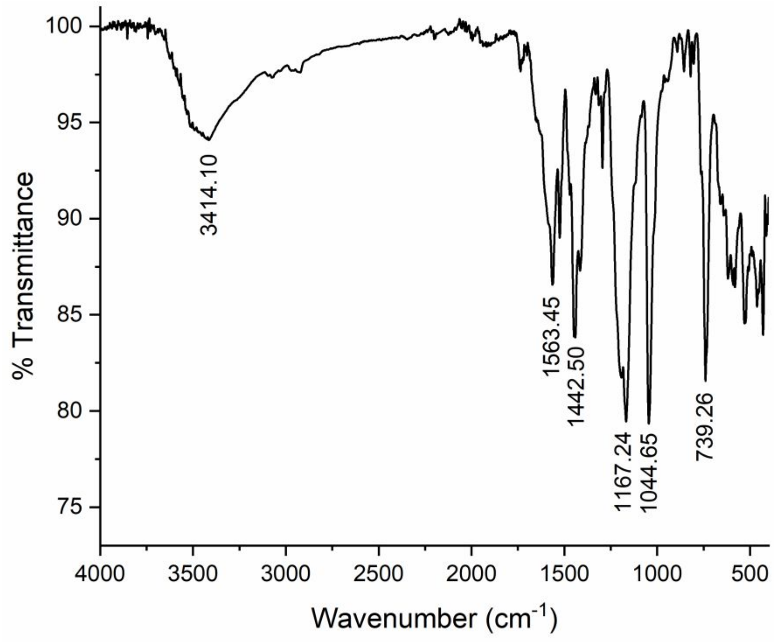

Figure S8. IR-ATR spectrum of 1.Cl.

3.*2.3 Electrospray Ionization Mass Spectroscopy ESI-MS of* **1.Cl**

[**1.Cl** – 2 K^+ + Na^+]$^-$: $C_{26}H_{23}ClN_4NaO_6PtS_2$

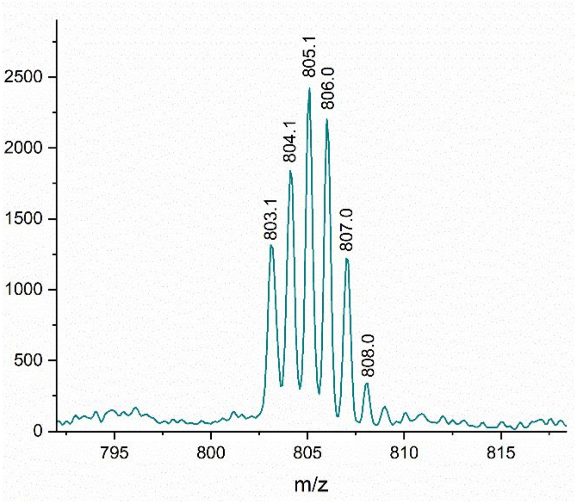

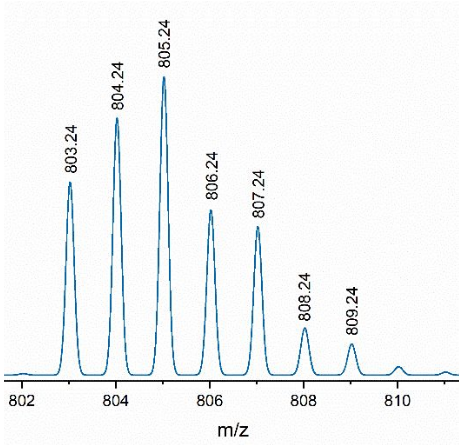

Experimental isotope distribution

Theoretical isotope distribution

Figure S9. Negative scan MS-ESI spectrum of **1.Cl** in CH_3OH.

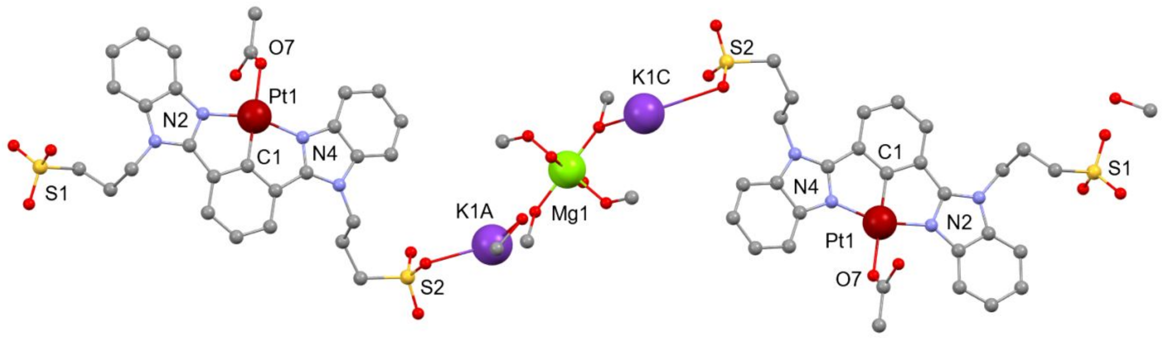

Figure S10. Perspective view of the unit cell of the 1.Aco crystal containing the two independent molecules and the counterions. Hydrogen atoms are omitted for clarity.

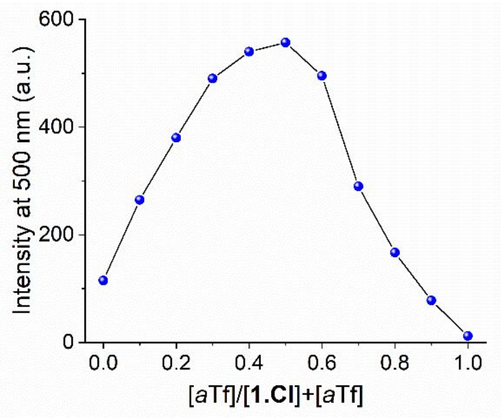

Figure S11. Job continuous variation plot of the *a*Tf-1.Cl supramolecular complex system at a final concentration of 5 mM in buffered water (10 mM HEPES, pH= 7.4).

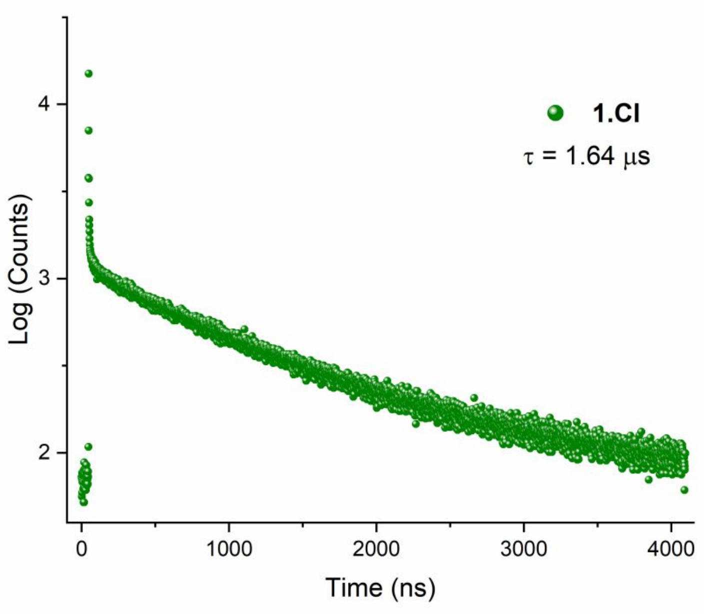

Figure S12. Luminescence decay profile (λ_{ex}= 355 nm) of aqueous solution of 1.Cl.

Lifetime measurements: A Time Correlated Single Photon Counting system coupled to a custom-built confocal microscope was used to acquire the fluorescence lifetimes. A 355 nm picosecond laser pulsed at 10 MHz (LDH-DC-405, PicoQuant) was focused into a 1 cm quartz cell with a 0.85 NA microscope objective. The fluorescence collected with the same objective passed through a 366 nm long-pass 27 dichroic mirror (Chroma T510lpxrxt), a 364 nm notch filter (Chroma ZET405nf), and a 425 nm long pass emission filter (Chroma ET425lp) and was focused to an avalanche photodiode (PD-050-CTE, MPD). The laser controller (PDL-800-D, PicoQuant) and the APD were connected to a TCSPC card (PicoHarp 300, PicoQuant). The power of irradiation was controlled to obtain less than 1.5 % of the detection events to avoid pile-up effects on the recorded histogram. Allura Red (analytical standard, Aldrich) was used to obtain the IRF under the same conditions of irradiation. All data were obtained and treated in SymphoTime 64 software (PicoQuant).

3. Thermodynamic parameter by Isothermal Titration Calorimetry

Thermodynamic parameters determined by 1.Cl titrated by *a*Tf by One binding site mode

T (K) Temperature

n binding correlation

K_b binding constant

K_d dissociation constant

ΔH_b molar binding enthalpy

ΔG_b molar binding free energy of Gibbs

$T\Delta S_b$ molar binding entropy multiplied by temperature

Table S5. Binding properties obtained from the calorimetric titration of 1.Cl over *a*Tf.

T (K)	η	K_b (x10^6 M^{-1})	K_d (x10^{-6} M)	ΔH_b (kcal/mol)	ΔG_b (kcal/mol)	$T\Delta S_b$ (kcal/mol)
298.15	0.79±0.01	3.38±0.53	0.30±0.05	-8.2±0.3	-8.9	-0.7

4. MALDI-TOF-MS

Antitumoral ligand, 1.Cl

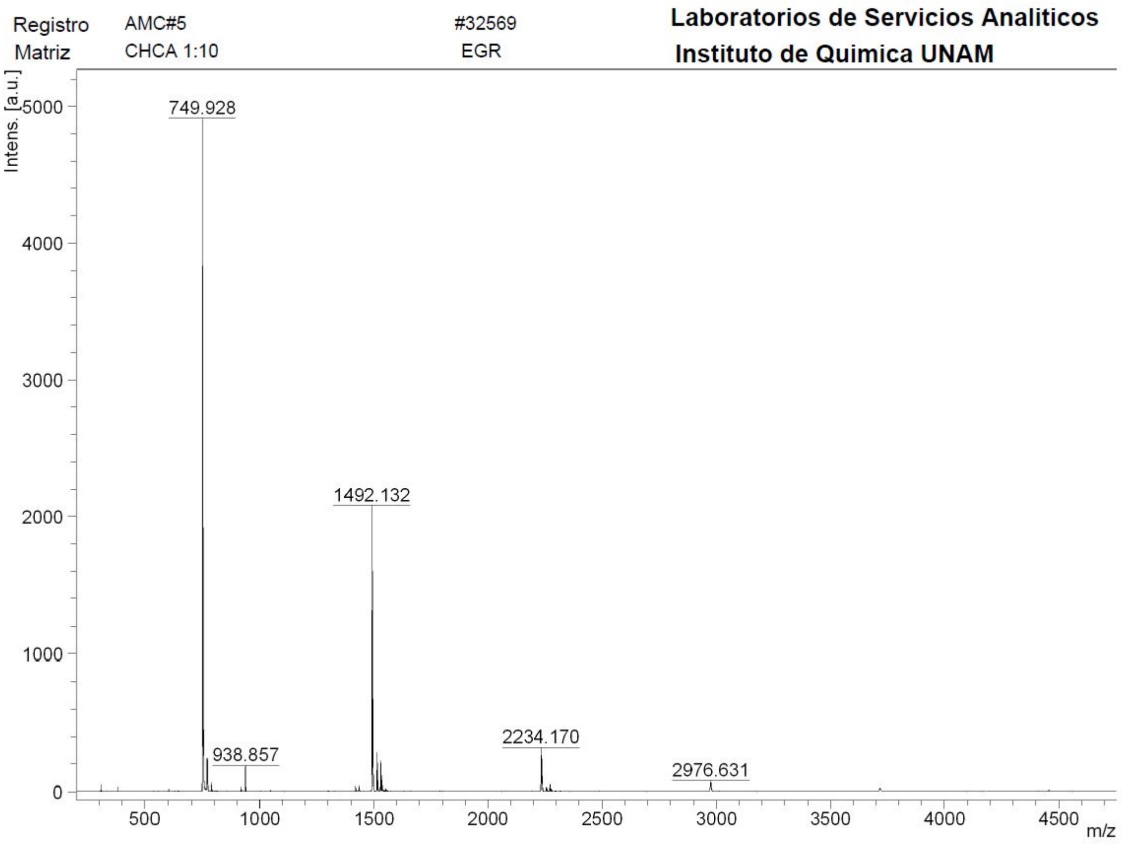

Acquisition Parameter

Date of acquisition	2025-03-13T15:55:16.296-06:00
Acquisition method name	D:\Methods\flexControlMethods\LNM_UNAM\RP_2465_ciclo BIEN.par
Aquisition operation mode	Reflector
Voltage polarity	POS
Number of shots	89
Name of spectrum used for calibration	
Calibration reference list used	PeptideCalibStandard mono_eren

Instrument Info

User	UNAM
Instrument	FLEX-PC
Instrument type	microflex

D:\data\LSA\32569\0_G8\1

Bruker Daltonics flexAnalysis printed: 3/13/2025 4:04:25 PM

*Human apo-transferrin, a*Tf

Registro AMC#1 #32307 **Laboratorios de Servicios Analiticos**

Matriz SA 1.5:5 EGR **Instituto de Quimica UNAM**

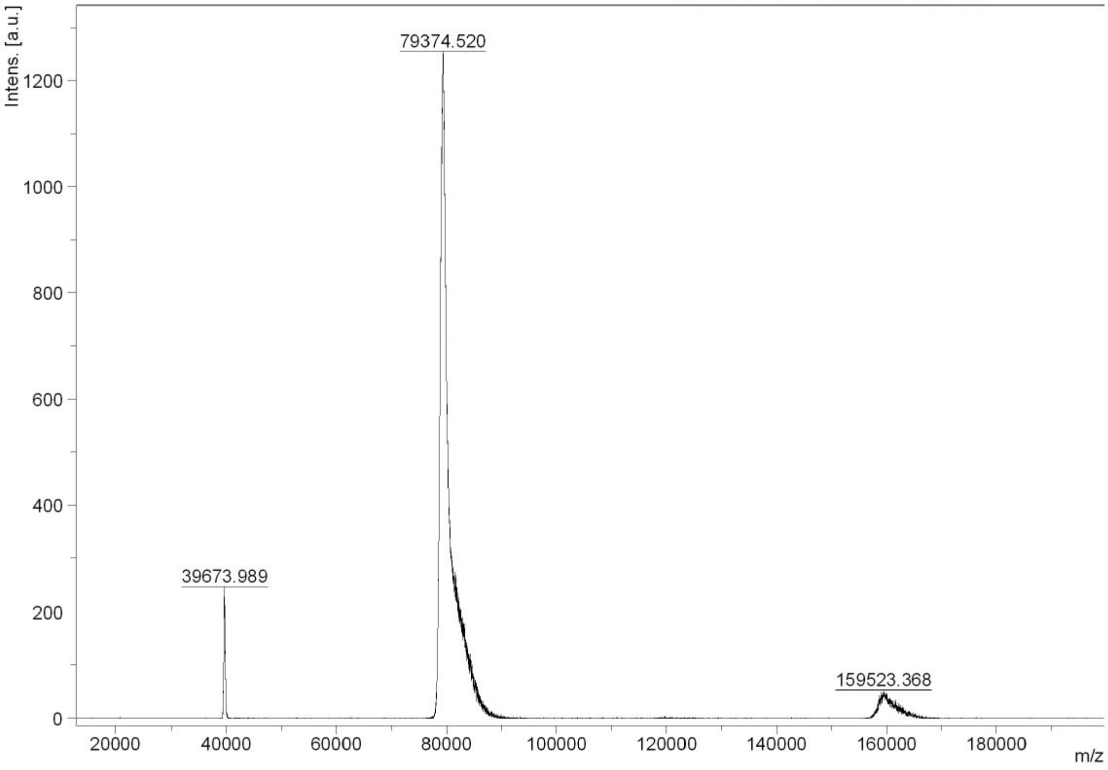

Acquisition Parameter

Date of acquisition	2025-03-04T18:24:00.562-06:00
Acquisition method name	D:\Methods\flexControlMethods\LNM_UNAM\LP_66kDa.par
Aquisition operation mode	Linear
Voltage polarity	POS
Number of shots	400
Name of spectrum used for calibration	
Calibration reference list used	Protein2CalibStandard_dimer eren

Instrument Info

User	UNAM
Instrument	FLEX-PC
Instrument type	microflex

D:\data\LSA\32307\0_E8\1

Bruker Daltonics flexAnalysis printed: 3/4/2025 7:03:12 PM
